# Ions‐Silica Percolated Ionic Dielectric Elastomer Actuator for Soft Robots

**DOI:** 10.1002/advs.202303838

**Published:** 2023-10-04

**Authors:** Hanbin Choi, Yongchan Kim, Seonho Kim, So Young Kim, Joo Sung Kim, Eseudeo Yun, Hyukmin Kweon, Vipin Amoli, U. Hyeok Choi, Hojin Lee, Do Hwan Kim

**Affiliations:** ^1^ Department of Chemical Engineering Hanyang University Seoul 04763 Republic of Korea; ^2^ School of Electronic Engineering Soongsil University Seoul 06978 Republic of Korea; ^3^ Department of Polymer Science and Engineering and Program in Environmental and Polymer Engineering Inha University Incheon 22212 Republic of Korea; ^4^ Department of Sciences and Humanities Rajiv Gandhi Institute of Petroleum Technology Amethi 229304 India; ^5^ Department of Intelligent Semiconductors Soongsil University Seoul 06978 Republic of Korea; ^6^ Institute of Nano Science and Technology Hanyang University Seoul 04763 Republic of Korea; ^7^ Clean‐Energy Research Institute Hanyang University Seoul 04763 Republic of Korea; ^8^ Present address: Hirosawa Thin Film Devices Laboratory RIKEN, 2‐1 Hirosawa Wako City, Saitama Prefecture 351‐0198 Japan

**Keywords:** electrode polarization, electromechanical conversion, ionic electroactive polymer actuator, ions‐silica percolated ionic dielectric elastomer, soft robot

## Abstract

Soft robotics systems are currently under development using ionic electroactive polymers (i‐EAP) as soft actuators for the human‐machine interface. However, this endeavor has been impeded by the dilemma of reconciling the competing demands of force and strain in i‐EAP actuators. Here, the authors present a novel design called “ions‐silica percolated ionic dielectric elastomer (i‐SPIDER)”, which exhibits ionic liquid‐confined silica microstructures that effectively resolve the chronic issue of conventional i‐EAP actuators. The i‐SPIDER actuator demonstrates remarkable electromechanical conversion capacity at low voltage, thanks to improved ion accumulation facilitated by interpreting electrode polarization at the electrolyte‐electrode interface. This approach concurrently enhances both strain (by approximately 1.52%) and force (by roughly 1.06 mN) even at low Young's modulus (merely 5.9 MPa). Additionally, by demonstrating arachnid‐inspired soft robots endowed with user‐desired tasks through control of various form factors, the development of soft robots using the i‐SPIDER that can concomitantly enhance strain and force holds promise as a compelling avenue for ushering in the next generation of miniaturized, low‐powered soft robotics.

## Graphical abstract

A material platform exhibiting ionic liquid‐confined silica microstructures that effectively resolve the chronic issue of conventional i‐EAP actuators is presented. This material‐based actuator showcases remarkable electromechanical conversion capacity at low voltage, thanks to improved ion accumulation facilitated by interpreting electrode polarization at the electrolyte‐electrode interface. The development of soft robots using are materials, which can concomitantly enhance strain and force, endow with user‐desired tasks through control of various form factors.

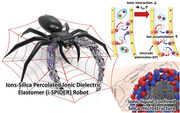

## 1 Introduction

Soft actuators play a critical role in enabling robots to perform desired operations and achieve directional locomotion in soft robotics, a new paradigm that offers innovative applications in medical science, exploration missions, and human assistance or augmentation.^[^ ^1^, ^2^, ^3^, ^4^ ^]^ Recent studies have focused on electrically‐driven polymer actuators for the development of fast and miniaturized soft robots, owing to their flexibility, ease of fabrication, and lightweight nature.^[^ ^5^, ^6^, ^7^ ^]^ As a result, researchers have extensively explored electroactive polymer (EAP) actuators and ionic EAP (i‐EAP) actuators to exploit these advantages. In principle, EAP actuators offer rapid response times and high electromechanical energy density compared to i‐EAP.^[^ ^8^, ^9^ ^]^ However, most EAP actuators developed so far exhibit low actuator strain and require high‐driving electric fields (> 100 MV/m), which limits their use to low‐powered, miniaturized soft robots designed for specific applications.^[^ ^10^, ^11^, ^12^ ^]^

On the other hand, i‐EAP actuators have gained considerable attention in soft robotics as artificial muscles, soft grippers, and for their soft tactile feedback due to their low‐power consumption, high durability, and low‐cost fabrication.^[^ ^13^, ^14^, ^15^ ^]^ However, their low electromechanical efficiency and relatively low blocking force impede their widespread applications (see Table S1, Supporting Information).^[^ ^5^, ^16^ ^]^ Thus, numerous approaches have been studied to improve their performance to achieve high strain and high blocking force simultaneously, as these two parameters must be secured in soft robotics among various actuation performances, such as strain, electromechanical efficiency, force, and durability.^[^ ^17^ ^]^

As an active part for i‐EAP actuators that mainly govern the actuation mechanism and performance, ionic polymers have been intensively explored to improve the performance of i‐EAP actuators by controlling the soft polymer matrix, ionic component, and conducting additives inside the ionic polymers. For example, actuator deformation is effectively enhanced by adjusting the type of ionic materials (e.g., common ionic liquids (ILs),^[^ ^18^ ^]^ single ion conductor,^[^ ^19^ ^]^ and zwitterion^[^ ^20^, ^21^ ^]^) and soft polymer matrix (e.g., elastomer,^[^ ^15^, ^22^ ^]^ polyvinylidene fluoride derivatives,^[^ ^23^, ^24^ ^]^ and Nafion^[^ ^25^ ^]^) or by introducing conducting additives, such as graphene^[^ ^26^, ^27^ ^]^ and carbon nanotubes,^[^ ^28^ ^]^ to increase the ionic conductivity of soft ionic polymers. Additionally, to achieve the actuation force required for soft robots, i‐EAP actuators with improved mechanical properties were developed by introducing inorganic particles into ionic polymers^[^ ^29^, ^30^ ^]^ or using metal electrodes.^[^ ^13^, ^18^, ^31^ ^]^ However, previous studies so far were limited to improving either force or strain, not both main performance indicators of the electrically driven soft actuator at the same time.

A primary cause contributing to this limit is that the mechanical property (Young's modulus) of the active layer has opposite effects on the two main performances, as can be derived from Maxwell's stress.^[^ ^32^, ^33^ ^]^ The high mechanical properties of the active layer, which is essential for improving the actuator force, unavoidably involve attenuation of the strain. Therefore, an ionic polymer actuator aiming for the enhancement of both force and strain should compensate for strain attenuation resulting from high mechanical properties by improving ionic properties, such as ionic conductivity and interfacial dielectric properties. For instance, Nan et al.^[^ ^34^ ^]^ presented i‐EAP actuators with improved performance using ionic polymer composites with enhanced ionic conductivity by introducing conductive fillers and ionic components into the rigid polymer. Wang et al.^[^ ^14^ ^]^ reported an ionic polymer with improved compatibility with ILs by modifying the ionic polymer matrix and developed i‐EAP actuators with high ionic conductivity. However, despite tremendous efforts in the prior studies to simultaneously improve the two crucial performances of the actuators by solely increasing the ionic conductivity, it is still extremely challenging to resolve their high stiffness (> 100 MPa) in realizing the high strain of i‐EAP actuators. This means that the ionic polymer matrix should reveal a mechanism to improve material limitations while retaining their essential properties to implement soft actuators with synergistic force and strain that can be applied to various soft robotic applications with a simple structure, but unexplored yet.

Here, we present an ion‐silica percolated ionic dielectric elastomer (i‐SPIDER)‐based soft actuator with improved strain and blocking force, showcasing its potential applications in low‐power soft robotics. The i‐SPIDER employs ion‐silica percolated microstructures uniformly embedded in a thermoplastic polyurethane (TPU) matrix, creating a synergistic architecture that enhances electrode polarization and improves both force and strain by manipulating the ionic interaction energy. Therefore, despite its low Young's modulus (5.9 MPa), the i‐SPIDER exhibits exceptional actuation performance, including large actuation strain (1.52%), high blocking force (1.06 mN), high strain rate (∼15.8% s^−1^), and high electromechanical energy density (∼687 J/m^3^). By exploiting the low binding energy of the ion pairs attached to the silica surface,^[35‒37]^ the i‐SPIDER enables the accumulation of more conducting ions, which in turn enhances the dielectric constant of the electrode polarization at the interface between the i‐SPIDER and the electrodes. Moreover, we have observed that augmenting ion accumulation not only improves the mechanical properties but also enhances the dielectric property, thereby boosting the overall actuator performance without compromising any other features. Consequently, we believe that the i‐SPIDER developed in this study can provide a platform for achieving both high actuation strain and blocking force, making it a promising approach for the next generation of miniaturized, low‐power soft robotics.

## 2 Results

### 2.1 Design Rule and Fabrication of i‐SPIDER for Soft Robots

**Figure 1:**
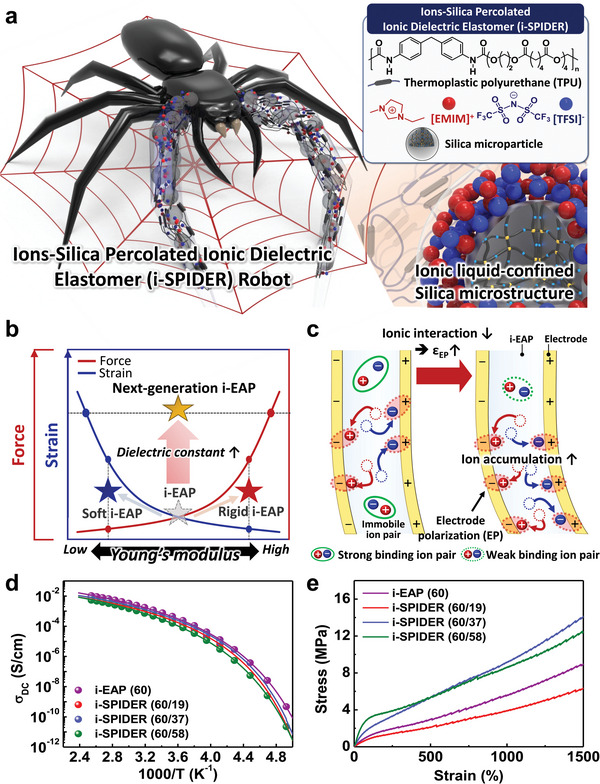
(a) Schematics of the arachnid‐inspired i‐SPIDER robot for soft robotic application. The top inset shows the chemical structures and schematic indexes of critical components (polymer matrix, IL, and silica microparticles) of i‐SPIDER films. The bottom inset shows the schematic depiction of IL‐confined silica microstructure as a crucial structure in i‐SPIDER films. (b) Conceptual schematics of the actuation performances (strain and force) comparing the soft, rigid, and next‐generation i‐EAP in terms of Young's modulus and dielectric constant. (c) Conceptual schematic illustration of the electrode polarization effect in i‐EAP with reduced ionic interaction for more ion accumulation leading to improved actuation performances. (d and e) Temperature‐dependent ionic conductivity (*σ_DC_*) and stress–strain relationship as a function of a series of i‐SPIDERs (60 wt% *Φ_IL_*) with different *Φ_SiO2_* loading.

**Figure** **1**a presents a schematic depiction of an arachnid‐inspired soft robot using an i‐SPIDER as a low‐voltage operating soft robotic interface. The designed i‐SPIDER, a hybrid version of conventional i‐EAP with a mixture of polymer matrix and ILs, contains IL‐confined silica microstructures that can locally confine ionic species inside the polymer matrix (inset in Figure 1a). For i‐SPIDER, the TPU was used as the main elastic matrix.^[^ ^38^ ^]^ In the IL‐confined microstructure, 1‐ethyl‐3‐methylimidazolium bis(trifluoromethane sulfonyl) imide ([EMIM]^+^[TFSI]^−^) and silica microparticles are loaded in the TPU matrix to form ionic confined regions, where ions are bonded on the surface of silica microstructures through hydrogen bonding interaction (discussed in detail later). To satisfy the performance of soft actuators required for implementing low‐power soft robots, material factors, such as ionic conductivity and mechanical properties of i‐EAP, are still being controlled, but the strain and force of the actuators exhibit a conflicting relationship (Figure 1b). For example, as soft i‐EAPs have improved ionic conductivity by plasticizing the polymer matrix with ionic additives,^[^ ^18^, ^19^ ^]^ the strain performance increases owing to the reduced Young's modulus, while their force decreases inevitably. Conversely, in the case of rigid i‐EAPs with improved Young's modulus, by introducing inorganic particles^[^ ^30^, ^34^ ^]^ or modifying the polymer matrix,^[^ ^14^ ^]^ these actuators can achieve high force performance, but the decreased strain is an unavoidable characteristic because Young's modulus directly and actively affects the actuation force and strain of electrically driven soft actuators. To overcome the limitations imposed by the mechanical properties of existing soft and rigid i‐EAPs, tailoring a dielectric constant, which can act synergistically on the performance of electrically driven soft actuators, is an ideal approach to realize next‐generation i‐EAPs. These next‐generation i‐EAPs can exhibit increased force and strain at lower voltages (see Note S1 for more details, Supporting Information).

In principle, the performance of i‐EAP actuators is affected by the interfacial dielectric constant (ε_EP_), which represents electrode polarization due to ion accumulation at the interface between the i‐EAP and electrode. Although ILs in typical i‐EAPs are reported to have a high degree of ion dissociation, the undissociated ion pairs exist when they are under a low electric field or low dielectric medium owing to the intrinsic ionic interaction of ion pairs.^[^ ^39^, ^40^ ^]^ As described in Figure 1c, weak‐binding ion pairs can more readily reduce the ionic interactions of the ion pairs, resulting in additional interfacial polarization with more ion accumulation at the interface, compared to strong‐binding ion pairs.^[^ ^41^ ^]^ Therefore, the increase in dielectric constant enables the development of next‐generation i‐EAP actuators that simultaneously improve strain and force by compensating for the conflicting effects of the mechanical properties of i‐EAP.

We fabricated a series of i‐SPIDERs with different IL and silica contents (*Φ_IL_* and *Φ_SiO2_*, respectively), as shown in Table S2 in the Supporting Information. These silica microparticles (*Φ_SiO2_* = 19, 37, and 58 wt%) were introduced into i‐EAP together with ILs (*Φ_IL_* = 60 wt%), hereafter referred to as i‐SPIDER (*Φ_IL_/Φ_SiO2_* = 60/19, 60/37, and 60/58). The morphological analysis of the i‐SPIDER films with different IL‐confined silica microstructure contents suggested that the monodispersed silica microstructures (<diameter> ≈ 15 µm) are well dispersed inside the i‐SPIDER films (Figure S1, Supporting Information). As reported previously, in the general case of hybrid ionic materials containing untreated inorganic fillers, the inorganic fillers are known to impede the migration of ions.^[^ ^42^, ^43^ ^]^ Interestingly, in our i‐SPIDERs (60 wt% *Φ_IL_*), the ionic conductivities are almost similar to that of silica microparticle‐free i‐EAP ([EMIM]^+^[TFSI]^−^ loaded TPU matrix to be used as reference) with the same ion concentration, as shown in Figure 1d. These ionic conductivity results are independent of the variation of the IL‐contained TPU matrix according to different silica contents, as inferred from the microstructural analysis of a series of i‐SPIDERs (see Note S2 and Figures S1‐4 for more details, Supporting Information). Figure 1e shows the mechanical properties of i‐EAP and i‐SPIDERs according to different silica concentrations at 60 wt% *Φ_IL_*. As the mechanical property of i‐SPIDER increases with the amount of silica microstructure, i‐SPIDER (60/58) exhibits Young's modulus 4 times higher than that of i‐EAP (60) (Figure S5, Supporting Information). These results suggest that the IL‐confined silica microstructures not only contribute to mechanical properties, which are similar to those of ordinary inorganic fillers, but also can reduce ionic interactions of ion pairs, which can compensate for the decrease in ionic conductivity. Obviously, the design of i‐SPIDER provides a synergistically favorable architecture that improves mechanical properties while maintaining electrical properties. From this point of view, i‐SPIDER is expected to give rise to the fabrication of actuators that can simultaneously improve actuation force without compromising strain performance.

### 2.2 Molecular Characterization of i‐SPIDER Film

**Figure 2:**
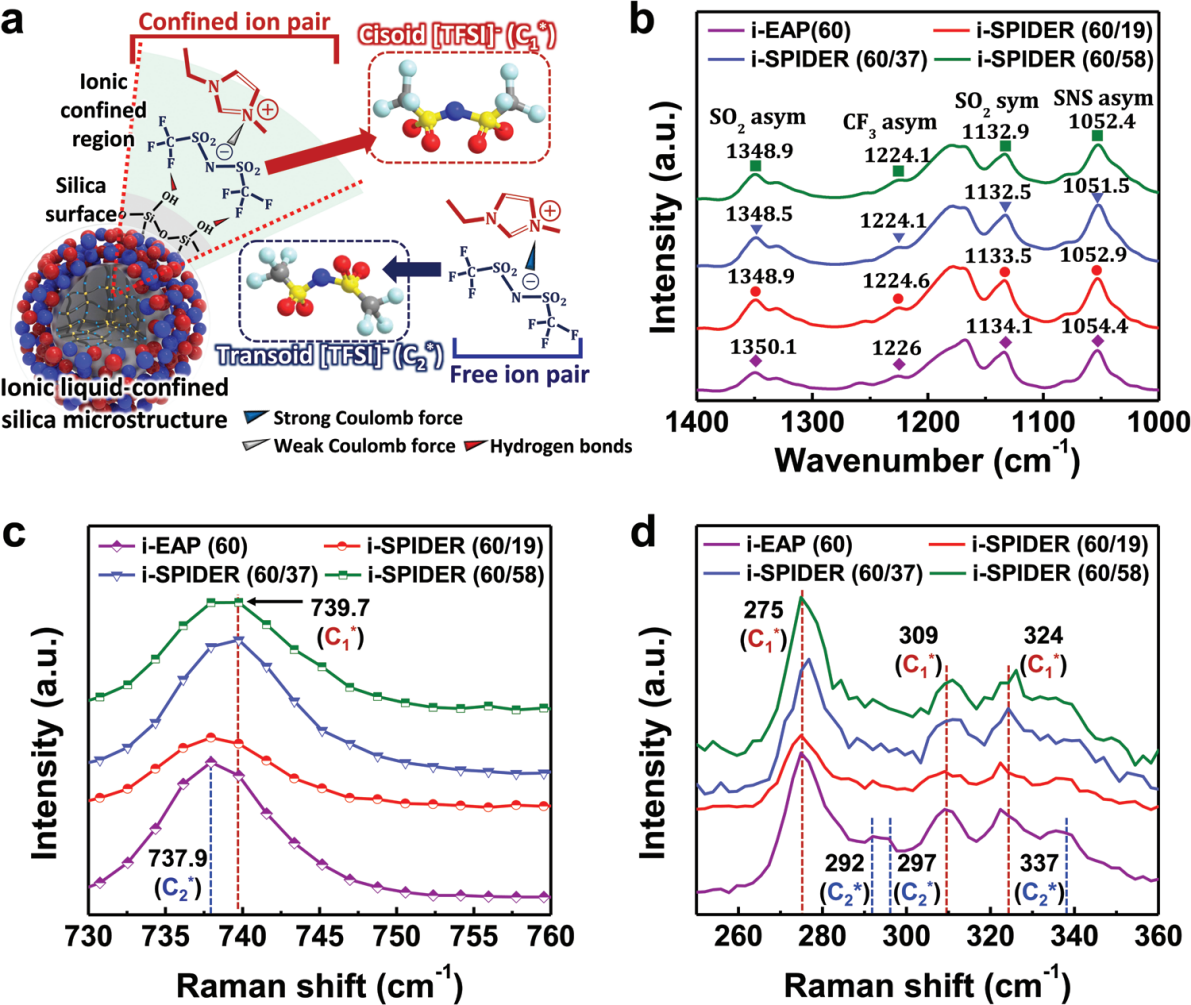
(a) Schematic depiction of IL‐confined silica microstructure with different conformations of [TFSI]^−^ anion at the ionic confined region on the silica surface and the free state in the matrix. The insets show the two conformations of [TFSI]^−^ anion as the cisoid structure (C_1_^*^) of the confined ion pair and the transoid structure (C_2_^*^) of the free ion pair. (b) FT‐IR spectra in the spectral region of 1400–1000 cm^−1^ (corresponding to [TFSI]^−^ anion stretching). (c) Raman spectra in the spectral range of 730–760 cm^−1^ (corresponding to [TFSI]^−^ anion expansion‐contraction mode). (d) The Raman spectra of conformational sensitive range 250–360 cm^−1^ of [TFSI]^−^ anion. The FT‐IR and Raman spectra results are shown for i‐EAP and i‐SPIDERs with different loadings of *Φ_SiO2_* (0, 19, 37, and 58 wt%). The content of IL is fixed (60 wt% *Φ_IL_*).

The designed IL‐confined silica microstructures for implementing the weak ionic interaction of ion pairs are composed of stepwise layers: i) hydrogen bonding‐governed silica surface and ii) Coulomb interaction‐governed ionic confined region (**Figure** **2**a). In [EMIM]^+^[TFSI]^−^, the [TFSI]^−^ anions naturally have two conformations, i.e., transoid (C_2_^*^) and cisoid (C_1_^*^) structures with relatively strong and weak Coulomb forces, respectively.^[^ ^35^, ^36^, ^37^ ^]^ In the bulk (free) state of [EMIM]^+^[TFSI]^−^, most [TFSI]^−^ anions exist in a thermodynamically stable transoid structure. However, a conformation change to the cisoid structure can be induced in specific (confined) regions when combined with external factors, such as the hydrogen bonding of silanol groups on the silica surface for implementing the weak ionic interaction of ion pairs.^[^ ^44^, ^45^ ^]^ Thus, the proposed specific structure was designed to realize weak ionic interaction that can improve electrode polarization through IL‐confined silica microstructures. Furthermore, Fourier transform infrared spectroscopy (FT‐IR) and Raman spectroscopy measurements were performed to confirm the formation of IL‐confined silica microstructure in the i‐SPIDER films, as illustrated schematically in Figure 2a. Figure 2b shows the FT‐IR spectra of the i‐EAP and i‐SPIDERs with different silica contents at the fixed 60 wt% *Φ_IL_*. Shifting of FT‐IR vibrational bands of SO_2_, CF_3_, and SNS groups of [TFSI]^−^ anion toward lower wavenumbers in i‐SPIDERs reveals the immobilization of ion pairs on the surface of silica microstructures due to the hydrogen bonding interaction with silica particles and ILs, as reported previously.^[44‒46]^

As the silica concentration increases from 0 to 58 wt%, the overall peak shift of the FT‐IR vibrational band of the [TFSI]^−^ anion suggests that more confined ion pairs are formed in the IL‐confined silica microstructures.^[^ ^47^, ^48^ ^]^ Figures 2c and 2d show the Raman spectra of the i‐EAP and i‐SPIDERs with varied silica concentrations at 60 wt% *Φ_IL_* obtained under identical experimental conditions. A pronounced blue shift in [TFSI]^−^ expansion‐contraction mode at ∼740 cm^−1^ in the i‐SPIDERs (60/37 and 60/58) films indicates the conformational change of the [TFSI]^−^ anion. Furthermore, the conformational change in [TFSI]^−^ anion was confirmed by obtaining low‐frequency Raman spectra (250–360 cm^−1^) sensitive to the conformation change. Interestingly, the transoid (C_2_^*^) conformers (292, 297, and 337 cm^−1^) dominate in the i‐EAP film (blue vertical dashed lines in Figure 2d), whereas in i‐SPIDER, cisoid (C_1_^*^) conformers (275, 309, and 324 cm^−1^) are also observed (red vertical dashed lines in Figure 2d). These results indicate that the well‐dispersed silica microparticles (as confirmed in Figure S1, Supporting Information) in the i‐SPIDERs constitute ionic confined regions (as depicted in Figure 2a), in which [TFSI]^−^ anions are confined on the surface of silica microparticles through hydrogen bonding interactions. It has been reported that the conformational change in ionic species in IL (as observed for [TFSI]^−^ in our case) can weaken Coulomb interactions between the ions, thus promoting ion dissociation.^[35‒37]^ In i‐SPIDER, hydrogen‐bonded [TFSI]^−^ anions with changed conformation (i.e., cisoid conformation) can weaken the Coulomb interaction between the [EMIM]^+^ cation and [TFSI]^−^ anion. These tendencies of i‐SPIDERs suggest that ion accumulation on the electrode interface can be further facilitated under an external electric field through improved ion transportation by IL‐confined silica microstructures.

### 2.3 Dielectric Spectroscopic Analysis of i‐SPIDER Film

To investigate the advantages of IL‐confined silica microstructure in terms of electrode polarization, we evaluated ion transportation and dielectric properties of i‐SPIDER by measuring ionic conductivity and dielectric constant as a function of frequency (*f*) and temperature. These natures of i‐SPIDER are important parameters that correlate with the overall actuation performances, such as force, strain, speed, and electromechanical energy density. Particularly, to confirm the expected improvement in ion accumulation of the proposed i‐SPIDER, it is essential to understand the dielectric relaxation characteristics for the dielectric loss derivative (*ε_der_*) and dielectric permittivity (*ε’*), which can determine the formation time and degree of electrode polarization, respectively (see Note S3, Figures S6‒S7, and Equations S2‒S4 for more details, Supporting Information). Based on the understanding of dielectric relaxation characteristics, we compared the overall dielectric relaxation characteristics to interpret the change in electrode polarization observed in i‐SPIDER with 60 wt% *Φ_IL_*.

**Figure 3:**
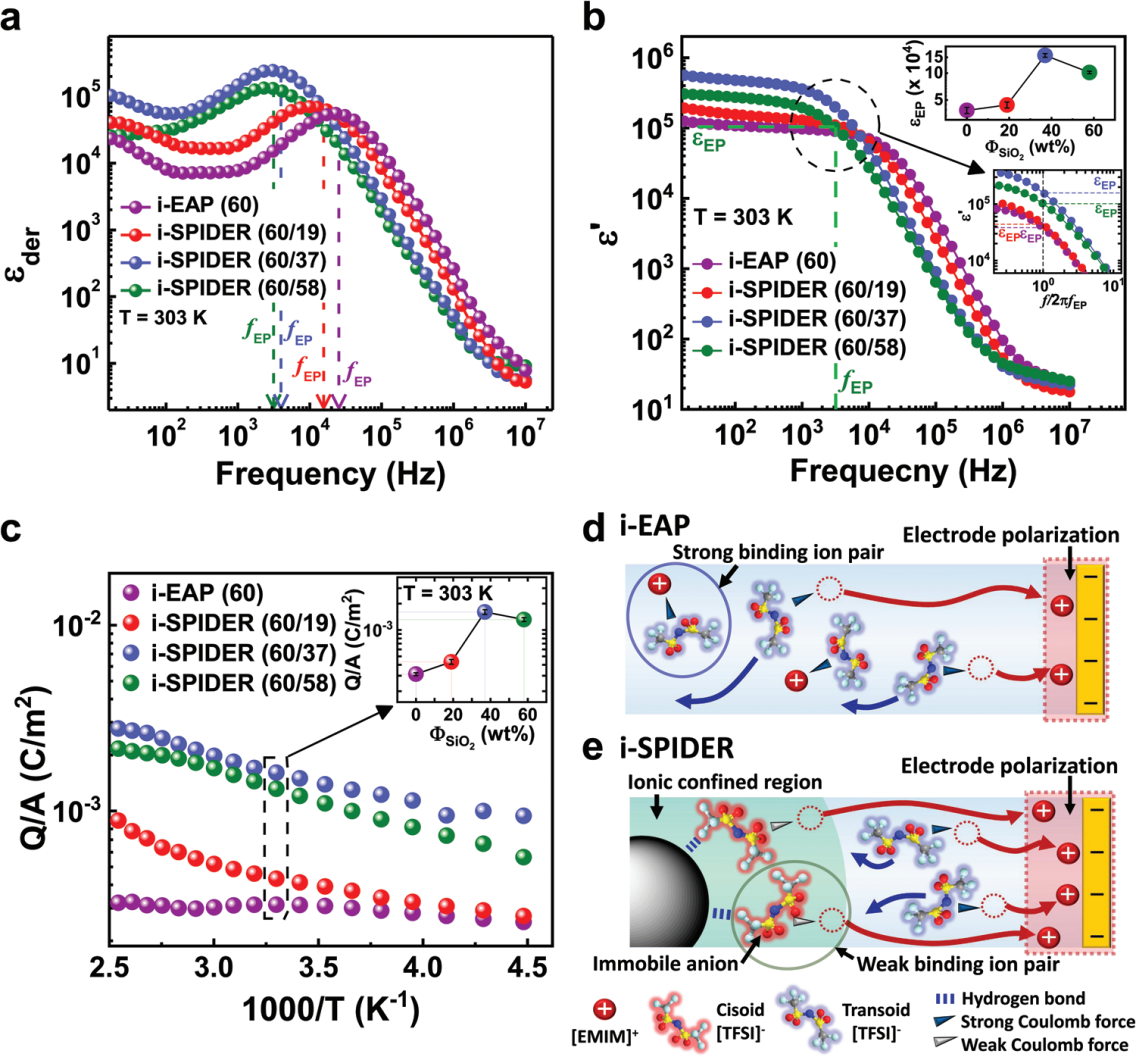
(a) Frequency dependence of *ε_der_* for i‐SPIDERs (60 wt% *Φ_IL_*) with various *Φ_SiO2_* and their neat i‐EAP (60) at 303 K along with *f_EP_*, indicated by dash arrows. (b) *ε’* as a function of frequency for i‐SPIDERs (60 wt% *Φ_IL_*) with various *Φ_SiO2_* and their neat i‐EAP (60) at 303 K. The dashed lines indicate *f_EP_* and *ε_EP_* of i‐SPIDER (60/58) after electrode polarization is complete. The bottom and top insets show *ε’* as a function of frequency multiplied by *f_EP_* (*ε’* versus *f/f_EP_* at the frequencies near *f/f_EP_* = 1) and *ε_EP_* as a function of *Φ_SiO2_*, respectively. Error bars indicate standard deviation. (c) Effect of silica content on temperature‐dependent charge density. The inset shows the charge density as a function of *Φ_SiO2_* at 303 K. Error bars indicate standard deviation. Schematic illustrations of the ion behaviors in (d) i‐EAP and (e) i‐SPIDER induced by increasing electrode polarization.

All electrode polarization frequencies (*f_EP_*) were calculated using the empirical modification of the Macdonald model (Figure S8 and Equation S5, Supporting Information). **Figure** **3**a displays *ε_der_* spectra of the i‐EAP and the i‐SPIDERs with different silica content at 60 wt% *Φ_IL_* obtained under identical experimental conditions. The *ε_der_* spectra of the i‐EAP and the i‐SPIDERs exhibit clear peaks between frequencies (*f_EP_* = 10^3^–10^5^ Hz), related to the electrode polarization process. It is important to note that electrode polarization (analogous to charging a capacitance) in i‐EAP (60) is fully developed at a higher frequency (*f_EP_* ∼ 2 × 10^4^ Hz) compared to i‐SPIDERs (60 wt% *Φ_IL_*) (*f_EP_* < 2 × 10^4^ Hz), indicated by vertical dashed arrows in Figure 3a. These observations indicate that transporting ions takes a longer time (i.e., the time scale of electrode polarization, *τ_EP_*) to accumulate near the electrodes to fully polarize the ions in i‐SPIDER (Figures S9 and S10a, Supporting Information). Additionally, for silica microparticle‐free i‐EAPs, the shortest *τ_EP_* (as observed for *Φ_IL_* = 60 wt%) indicates fast ion migration, also reflected in an increase in ionic conductivity (*σ_DC_*) with the IL content (Figure S10b, Supporting Information). However, this does not seem to be the case for the IL‐confined silica microstructure‐based i‐SPIDERs (with varying silica contents at the fixed 60 wt% *Φ_IL_*) because they have almost similar *σ_DC_* as their neat i‐EAP (60) (Figure 1d). The other plausible reason is that *τ_EP_* can directly increase if more transporting ions can participate in the electrode polarization, which is an ion accumulation process, possibly because the confined ion pairs with weak Coulomb interactions in the IL‐confined silica microstructure readily respond to an applied electric field.

To establish such an ion accumulation phenomenon at the electrode, the frequency dependence of *ε’* was also explored, as shown in Figure 3b, which allows us to estimate the dielectric constant of electrode polarization (*ε_EP_* = *ε’*(*f EP*)) at the frequency when electrode polarization is complete (dashed lines in Figure 3b and its bottom inset). i‐SPIDERs with higher silica content (37, 58 wt% *Φ_SiO2_* at 60 wt% *Φ_IL_*) have almost two times higher *ε_EP_* than the silica microparticle‐free i‐EAP (60) and i‐SPIDER (60/19) (top inset of Figure 3b), suggesting that i‐SPIDERs have more transporting ions that accumulate near the electrode owing to hydrogen bonding‐driven complexation of the IL‐confined silica microstructures, which reduce the binding energy of ion pairs.^[35‒37]^ This result significantly increases ε’ and simultaneously forms an electric double layer at each electrode. Therefore, the build‐up of migrating ions in the Stern layer can control actuation performance. Furthermore, when the i‐SPIDER actuator is analytically modeled as a simple equivalent resistor–capacitor circuit, the correlation between charge density and *τ_EP_* can be considered (see Note S3 and Equation S3, Supporting Information). Then, the simple relationship for the charge accumulation per unit electrode surface area (charge density, *Q/A*) can be obtained using the following equation:^[^ ^49^, ^50^ ^]^where *Q* is the charge within the Stern layer, *C* and *R* are the capacitance and resistance of the equivalent circuit, respectively, *V* is the applied voltage, *A* is the surface area of the electrode, *L* is the thickness of the active layer, and *ε_0_* is the vacuum permittivity. Thus, the charge density at the electrode can be determined by the product of the material properties of *τ_EP/_L* and *σ_DC_* for the applied voltage. Moreover, the charge density can be expressed as *ε_EP_* because *τ_EP_* is correlated with *ε_EP_* and *σ_DC_* as *τ_EP_* = *ε_EP_ε_0_/σ_DC_*.^[^ ^51^, ^52^ ^]^ Figure 3c and Figure S10c in the Supporting Information show the charge densities of a series of i‐SPIDERs at the electrode. These charge densities were calculated using Equation (1) as a function of *Φ_SiO2_* (see the top inset of Figure 3b). i‐EAP (60) has the smallest charge density among a series of i‐SPIDERs with 60 wt% *Φ_IL_*, consistent with the observation of the shortest *τ_EP_* (Figures S9 and S11, Supporting Information) and smallest *ε_EP_* (Figure 3b). On the other hand, higher charge densities were observed in i‐SPIDERs with 37 and 58 wt% *Φ_SiO2_* at 60 wt% *Φ_IL_*, which have a higher *ε_EP_* (see Figure 3b and its top inset) and longer *τ_EP_* (Figures S9 and S11, Supporting Information) compared to that of i‐EAP (60). This enhancement in charge density was also partially observed in i‐SPIDER with lower IL content (Figure S10c, Supporting Information). Therefore, the property of higher *ε_EP_* appears in i‐SPIDERs containing a high content of IL‐confined silica microstructure, which leads to more ion accumulation (such as an increase in charge density) over an extended time for complete electrode polarization.

### 2.4 Operating Mechanism of i‐SPIDER Actuator

As can be compared in Figure 3d and 3e, these characteristics are expected to be derived from the IL‐confined silica microstructure, a unique structure of i‐SPIDER films. An applied external electric field dissociates the free ion pairs in i‐EAP films to form neat electrode polarization at the electrode. Nevertheless, some undissociated electrically neutral ion pairs with relatively strong Coulomb interactions partially exist concurrently within i‐EAP films (Figure 3d), and these ions cannot participate in the polarization near the electrode. In contrast, in the case of i‐SPIDER, the IL‐confined silica microstructure induces another polarization by solely [EMIM]^+^ cation migration under an electric field, in addition to the polarization by the dissociated ion pairs observed in i‐EAP (Figure 3e). This is presumably caused by the thermodynamically unstable ionic interaction (relatively weak Coulomb interaction) of the [EMIM]^+^ cation with the cisoid [TFSI]^−^ anchored to the silica surface by hydrogen bonds. These results show enhanced ion accumulation in pure ionic liquid with higher ionicity, reflecting the degree of ionic dissociation arising from the ionic interaction of the ionic liquids (Figure S12).^[^ ^39^ ^]^ Furthermore, an increase in silica content in a series of i‐SPIDER with 60 wt% *Φ_IL_* leads to improved capacitance. This implies that reducing the ionic interaction of ion pairs (cisoid [TFSI]^−^ based ion pairs), which are present in i‐SPIDER films, increases electrode polarization and induces more ion accumulation at the interface between the electrode and i‐SPIDER films. The marked enhancement in the dielectric polarization by facilitating ion dissociation can simultaneously improve the actuator strain and force.

### 2.5 i‐SPIDER Actuator Performance

**Figure 4:**
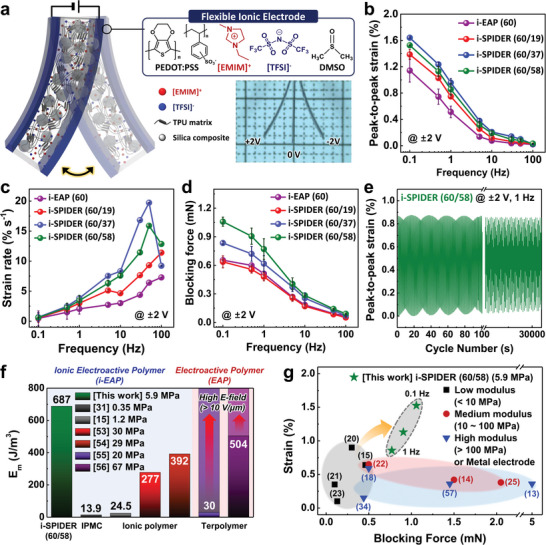
(a) Schematic depiction of the bending performance of the i‐SPIDER actuator. The top and bottom insets show the chemical structures consisting of a flexible ionic electrode and the optical image of the bending performance of i‐SPIDER (60/58) actuator under ±2 V DC bias. (b and c) Frequency dependency of peak‐to‐peak bending strain and strain rate of actuators using a series of i‐SPIDER (60 wt% *Φ_IL_*) at ±2 V AC bias. Error bars represent standard deviation. (d) Blocking force responses of a series of i‐SPIDER actuators with 60 wt% *Φ_IL_* when applying ±2 V AC bias. Error bars indicate standard deviation. (e) Reliability of i‐SPIDER (60/58) actuators under continuous operation in an ambient condition at ±2 V and 1 Hz. (f) Comparison of the strain‐derived *E_m_* of electrically driven soft actuators using the various i‐EAP materials (i‐SPIDER, IPMC, and ion gels) and EAP materials (terpolymer) at 10 mV/µm. The bright color bars of terpolymers show their *E_m_* at a high electric field (over 10 V/µm). (g) Comparisons of the blocking force–strain relationship between the actuators using i‐SPIDER (60/58) and the other i‐EAP materials in Young's modulus ranges; black area: soft modulus (< 10 MPa), red area: medium modulus (10–100 MPa), blue area: high modulus (> 100 MPa).

To evaluate actuator performance due to the enhanced electrode polarization effect, low‐voltage driven soft actuators were fabricated by spray coating electrode solutions on both sides of a series of i‐SPIDER films with 60 wt% *Φ_IL_*. **Figure** **4**a and Figure S13 in the Supporting Information show the schematic and photographs of the bending motion of an i‐SPIDER actuator with flexible ionic electrodes, comprising poly(3,4‐ethylene dioxythiophene):poly(styrene sulfonate) (PEDOT:PSS) introduced with [EMIM]^+^[TFSI]^−^ and dimethyl sulfoxide (DMSO) as hybrid additives. The flexible ionic electrode using the same ionic constituent of the active layer forms a well‐defined topology at the interface between the active layer and electrode, leading to optimized actuation performance.^[^ ^15^ ^]^

Figure 4b and Figure S14 in the Supporting Information show the frequency‐dependent strain and displacement of the i‐SPIDER actuators with different *Φ_SiO2_*, as examined at an input AC bias of ±2 V. In the strain performance considering the dimension of the films, the i‐SPIDER actuators demonstrated excellent actuation performance when changing the voltage‐switching frequencies from 0.1 Hz to 100 Hz. As the *Φ_SiO2_* increased, a series of i‐SPIDER actuators exhibited higher strain than that of i‐EAP (60) actuator. Particularly, the peak‐to‐peak strain of the i‐SPIDER (60/58) actuator was found to be similar to that of i‐SPIDER (60/37) even though the mechanical properties were more than twice as high. These trends are consistent with the result of the calculated charge density (Figure 3c), even though the peak‐to‐peak strain of the actuator tended to decrease with increasing frequency for a series of i‐SPIDER actuators, possibly due to insufficient time for ion diffusion in typical i‐EAP actuators. Nevertheless, the increase in strain rate with increasing frequency suggests that high‐speed operation of i‐SPIDER actuators is possible (Figure 4c). The strain rate of i‐SPIDERs with 37 and 58 wt% *Φ_SiO2_* is superior to that of other actuators, especially at high frequencies above 10 Hz (∼15.8% s^−1^). Figure 4d shows the blocking force of i‐SPIDER actuators under the excitation of 2 V AC bias in the frequency range of 0.1–100 Hz. Particularly, the blocking force of the i‐SPIDER (60/58) actuator is 1.05 mN, higher than that of the i‐EAP (60) actuator (0.65 mN) at 0.1 Hz. Moreover, the i‐SPIDER (60/58) actuator maintained durable strain performance without any apparent decrease in bending motion even during operation of over 30000 cycles at ±2 V and 1 Hz (Figure 4e). These results indicate that the IL‐confined silica microstructures in i‐SPIDER films simultaneously improve the two main actuator performances by providing an efficient ion transfer pathway and ion accumulation while achieving rapid bending motion and long‐term durability.

The characteristics of converting electrical energy into mechanical work can be evaluated by electromechanical energy density (*E_m_*), which is a factor comparing the soft actuators.^[^ ^32^, ^33^ ^]^ In this regard, the high *E_m_* of the i‐EAP actuator is required even with a low electrical source to realize low‐voltage driven soft robotics. Figure S15 in the Supporting Information shows the electromechanical energy density (*E_m_*) calculated from the strain performance of a series of i‐SPIDER actuators using Equation S1 using mechanical modulus and mechanical work (strain). Among a series of i‐SPIDER with 60 wt% *Φ_IL_*, the actuator using i‐SPIDER (60/58) with higher mechanical modulus and strain performance exhibited the highest *E_m_* in the overall frequency range. Additionally, we compared the *E_m_* of soft actuators based on several i‐EAPs, such as an ionic polymer‐metal composite (IPMC) and ionic polymer,^[^ ^15^, ^31^, ^53^, ^54^ ^]^ which have reported high strain performance with a low modulus at low voltage (Figure 4f). The i‐SPIDER (60/58) actuator showed an *E_m_* (687 J/m^3^), which is much higher than that of i‐EAP actuators (less than 400 J/m^3^) with low modulus of under 10 MPa. Furthermore, the improved *E_m_* of our actuator was similar to or even higher than that of EAP actuators based on terpolymer that is well‐known to have intrinsically high *E_m_*.^[^ ^55^, ^56^ ^]^ Generally EAP actuators have high *E_m_* require operating voltage lareger than 10 V. However, we found that the i‐SPIDER(60/58) actuator could achieve high *E_m_* of 687 J/m^3^ only at 10 mV, which confirms its high energy conversion characteristics, their common operating condition (more than 10^3^ J/m^3^ @ over 10 V/µm), highly indicating that the capability to utilize the electrical energy as mechanical energy is more efficient.

Figure 4g and Table S3 show a comparison between the blocking force and strain between low‐voltage driven soft actuators using i‐SPIDER (60/58) and other i‐EAP materials in terms of the mechanical modulus.^[^ ^13^, ^14^, ^15^, ^18^, ^20^, ^21^, ^22^, ^23^, ^25^, ^34^, ^57^ ^]^ The mechanical modulus of other i‐EAP materials was classified into three sections (low, medium, and high modulus) based on the modulus of the skeletal muscle used for comparison in studies of soft actuators for soft robotic applications.^[^ ^1^, ^58^ ^]^ In contrast to the low strain (< 1%) of previously reported soft actuators, which focused on improving the blocking force by using i‐EAPs with higher modulus, the i‐SPIDER actuator simultaneously improved two significant actuator performances. More notably, even at a low modulus (5.9 MPa) of i‐SPIDER (60/58), we confirmed that the blocking force (1.06 mN) and strain (1.5%) of our soft actuator were superior to those of the soft actuator using the general i‐EAP with soft modulus. Thus, the proposed actuator, which exhibits enhancement of the two main actuator performances based on high *E_m_* despite their soft mechanical properties under low operating voltage conditions, is suitable for future flexible and miniaturized robotic applications.^[^ ^11^, ^12^ ^]^

### 2.6 Arachnid‐Inspired Multifunctional Soft Robots using i‐SPIDER Actuator

**Figure 5:**
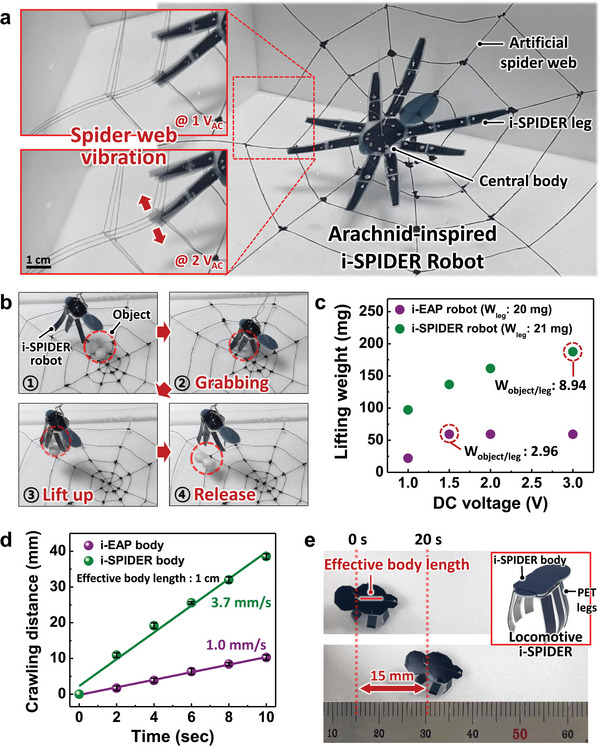
(a) A photograph showing an arachnid‐inspired i‐SPIDER robot sitting on an artificial spider web. The insets demonstrate the vibration motion of the spider web caused by the movement of i‐SPIDER actuator‐based spider legs with applied AC biases of ±1 V and ±2 V at 0.1 Hz. The scale bar is 1 cm. (b) Photographs of the i‐SPIDER robot during a series of object transfers, including grabbing, lifting up, and releasing objects. (c) Lifting performance of soft robots based on i‐EAP and i‐SPIDER with increasing DC voltages. The i‐EAP robot grabbed and lifted the the objects consisting of 1 or 2 Styrofoam balls while the i‐SPIDER robot grabbed and lifted the objects consisting of up to 7 Styrofoam balls. (d) Crawling distance of soft crawling robots using i‐EAP and i‐SPIDER body as a function of time at an AC bias of ±2 V and 1 Hz. The effective body length of the soft crawling robots is 1 cm. (e) Photographs of the crawling motion of the locomotive i‐SPIDER with a 5 mm effective body length. The inset shows the composition of the locomotive i‐SPIDER consisting of an i‐SPIDER body and PET legs.

To demonstrate the proof of concept and potential applications of the proposed i‐SPIDER (60/58) actuator in soft robotics, we implemented arachnid‐inspired soft robots capable of performing various user‐desired tasks. In order to fabricate an i‐SPIDER robot in the form of a spider, we introduced the patterned flexible ionic electrodes on the i‐SPIDER (**Figure** **5**a). The i‐SPIDER robot consists of eight legs connected to a central body via wires, allowing for individual operation of each i‐SPIDER leg (Movie S1, Supporting Information). When the i‐SPIDER robot is placed on an artificial spider web, the movement of the robot's legs causes the web to vibrate (Movie S2, Supporting Information). The movement of the i‐SPIDER legs can control the vibration amplitude of the spider web by adjusting the applied voltage. This enables the replication of spider‐specific and delicate behaviors, such as cleaning the web or communicating through the vibration of the spider web.^[^ ^59^, ^60^ ^]^ In addition, we demonstrated arachnid‐inspired soft robots optimized for user‐desired tasks by controlling various form factors using an efficient methodology based on the i‐SPIDER actuator.

As shown in Figure 5b and Movie S3 (Supporting Information), our i‐SPIDER robot with different leg angles could transfer an object made of balls placed on the artificial spider web while stably holding the object in place with a 2 V DC bias applied. The object could then be released by applying the opposite voltage when the desired location was reached. The weight‐lifting performance of the i‐SPIDER robot is superior to that of the i‐EAP (60) robot (Figure 5c, Figure S16, and Movie S3 Supporting Information). Specifically, when the object was approximately three times as heavy as the actuator constituting the legs of the soft robot, the i‐EAP robot failed to hold the object, and the object slid from the leg. On the other hand, the i‐SPIDER robot was able to grab and hold the object continuously. This improved lifting performance was attributed to the much higher force of the i‐SPIDER actuator. Furthermore, the i‐SPIDER robot shows a higher lifting weight ratio (ratio of leg mass to object mass) than the i‐EAP robot at increasing applied voltages, with a lifting weight ratio of 8.94 observed when the voltage was increased. In contrast, the maximum lifting weight ratio of the i‐EAP robot was saturated at a ratio of 2.96 at 1.5 V, whereas the ratio of the i‐SPIDER robot at 1 V appeared to be higher than the saturated ratio. These results suggest that i‐SPIDER has a favorable architecture that can overcome the effect of external bias, which generally contributes significantly to the performance of ionic actuators. This lifting weight ratio of the i‐SPIDER robot is higher than that reported previously using various ionic polymers.^[^ ^61^ ^]^

Moreover, we implemented movement characteristics of crawling insects by fabricating miniature robots (Figure S17a, Supporting Information).^[^ ^62^, ^63^ ^]^ To compare the crawling distance, we used both the i‐EAP and i‐SPIDER as bodies with the same effective body length of the actuator, which can directly affect the crawling motion. The crawling robot using the i‐SPIDER body moved forward by 37 mm in 10 seconds with a locomotion speed approximately 3.7 times faster than that of the i‐EAP body under the same time (Figure 5d, Figure S17b, and Movie S4, Supporting Information). The i‐SPIDER robot showed a superior crawling speed compared to other ionic polymer‐based miniaturized robots^[^ ^64^ ^]^, indicating its efficient control over soft robot movement speed compared to previous ionic polymers. Moreover, we observed successful locomotion of the i‐SPIDER even with different body and leg shapes from a miniature spider robot (Figure 5e and Movie S5). These demonstrations imply that our i‐SPIDER can satisfy various form factors and can be used successfully for low‐power soft miniature robotics.

## 3 Conclusion

We successfully demonstrated that i‐SPIDER containing IL‐confined silica microstructures shows simultaneous improvement in both force and strain of electrically driven soft actuators by overcoming the chronic conflicting inverse relationship between them. Notably, the key to realizing a unique design that improves two characteristics in this system is the introduction of an IL‐confined region in the TPU matrix, which plays a crucial role in electrode polarization for enhancing the electromechanical energy conversion in i‐SPIDER. This unique design enables the device to exhibit high mechanical properties as well as additional ion accumulation through the enhanced electrode polarization, facilitating the realization of superior actuator performances without any reduction in actuator strain and force. Therefore, despite the considerably low mechanical property (5.9 MPa) of i‐SPIDER, the proposed soft actuators simultaneously achieved the bending strain (∼1.52%) and blocking force (∼1.06 mN) based on high electromechanical energy density at a low voltage (2 V). The mechanism for these unprecedented actuation characteristics has been proven by detailed analysis of the electrode polarization effect while enhancing ion accumulation at the interface between the i‐SPIDER film and electrodes by altering the ionic interactions. We successfully exploited these actuation characteristics to demonstrate arachnid‐inspired robotic applications, such as mimicking insect behavior, transferring objects stably, and achieving faster crawling locomotion. This synergistically favorable design, which increases both strain and force properties, is expected to be effective in implementing next‐generation, low‐power, miniaturized soft robotics.

## 4 Experimental Section

### Chemicals and Materials

Tetraethyl orthosilicate (TEOS) and N,N‐dimethylformamide (DMF; 99.8%) were purchased from Sigma‐Aldrich. TPU beads were purchased from Kolon Industries, Inc. (product number: KA‐480). All these chemicals were stored in a glove box. 1‐ethyl‐3‐methylimidazolium bis(trifluoromethanesulfonyl)imide ([EMIM]^+^[TFSI]^−^; 99.9%) was purchased from Solvionic (Toulouse, France). Before usage, IL was stored under vacuum overnight to remove any residual moisture. Poly(3,4‐ethylenedioxythiophene):poly(styrene sulfonate) (PEDOT:PSS; PH1000) was purchased from Ossila Ltd. DMSO (99%) was purchased from Junsei Chemical Co. Ltd.

### Preparation of i‐SPIDER Film

Preparation of i‐SPIDER films, including IL‐confined silica microstructure, involves a sequential process: (i) preparation of IL‐confined silica microstructures via sol‐gel synthesis; (ii) preparation of TPU precursor solution; and (iii) preparation of TPU ternary component solution in which IL‐confined silica microstructures are dispersed, followed by an optimized heat‐treatment process to develop i‐SPIDER films. In the first step, the precursor solution for the IL‐confined silica microstructures was prepared by adding a suitable amount of TEOS in distilled water (0.25 mL) under continuous stirring at 40 °C for 10 min. Then, an appropriate amount of [EMIM]^+^[TFSI]^−^ was added dropwise to the TEOS–water mixture and stirred for 15 min at the same temperature. Precursor solutions for i‐SPIDER films with different contents of the IL‐confined silica microstructures were prepared by the same approach for different weight compositions of TEOS and [EMIM]^+^[TFSI]^−^ (see Table S2, Supporting Information). Then, 0.06 M hydrochloric acid (0.05 mL) was added dropwise to this mixture under the same stirring conditions, and the mixture was further stirred for 20 min. During the stirring process, the mixture gradually becomes turbid, indicating the hydrolysis/condensation of TEOS to form the silica network and the formation of IL‐confined silica microstructure via the interaction of ion species with the silica network. The obtained IL‐confined silica microstructure dispersion was further stirred for 10 min at 40 °C and then used for the next step. In the second step, the TPU precursor solution was prepared by dissolving TPU beads in DMF at a mass ratio of 1:5. The solution was stirred at 80 °C for 3 h. In the third step, IL‐confined silica microstructure dispersion (obtained in the first step) was added to the TPU precursor solution (obtained in the second step) dropwise under continuous stirring at 80 °C, and the resulting mixture was further stirred at 80 °C for another 24 h. To obtain i‐SPIDER film of the desired thickness (200 µm), a fixed amount of TPU ternary component solution was poured into a Teflon dish and heat treated at 80 °C for 72 h to remove the residual solvent completely. The weight percentage of ILs and silica microparticles reported in this work stands for the weight ratio of IL to IL + TPU and TEOS to TEOS + TPU, respectively. Various i‐SPIDER films with varying contents of IL (*Φ_IL_* = 20, 40, and 60 wt%) and silica contents (*Φ_SiO2_* = 19, 37, and 58 wt%) were prepared based on these weight ratios under identical reaction conditions. For reference, silica microparticle‐free i‐EAP films with different IL contents (*Φ_IL_* = 20, 40, and 60 wt%) were prepared using the ionic TPU precursor solution in which TPU beads and [EMIM]^+^[TFSI]^−^ were dissolved in DMF (a mass ratio of 1:3) under the same stirred conditions.

### Fabrication of i‐SPIDER Actuator using Ionic PEDOT:PSS Electrode

The i‐SPIDER actuators were fabricated using spray‐coating of ionic PEDOT:PSS solution on both sides of the i‐SPIDER films. First, the ionic PEDOT:PSS solution was prepared by dissolving PEDOT:PSS into distilled water with DMSO and [EMIM]^+^[TFSI]^−^ as additives. The weight composition of this solution was PEDOT:PSS:DMSO:[EMIM]^+^[TFSI]^−^:water = 1:0.05:0.015:3 (wt%). The mixed solution was stirred for 3 h and spray‐coated onto both sides of the i‐SPIDER films at 120 °C, followed by air‐condition annealing (47 900 Furnace, Barnstead Thermolyne Corp.) at 160 °C. Finally, after cutting the i‐SPIDER films, the soft actuator shape (length: 10 mm, width: 2 mm) was examined using an Olympus microscope equipped with a charge‐coupled device (CCD) camera (IMT i‐solution).

### Physical Characterization

The mechanical properties of all polymeric films were measured using a universal testing machine (Instron UTM 5569) in accordance with the ASTM D638 standard. The surface morphological features of a series of i‐SPIDER films were obtained using field‐emission scanning electron microscopy (FE‐SEM, JSM‐7800F Prime, JEOL Ltd.) equipped with a QUANTAX EDX system for elemental analysis. On‐specular X‐ray diffraction experiments were performed using a Bruker D2 Phaser desktop X‐ray diffractometer operating at 30 kV (10 mA) with a Cu K_α_ radiation source. The diffraction curve was acquired using a 5°–60° 2θ range for different types of i‐EAP and i‐SPIDER films with a thickness of 200 µm. Differential scanning calorimetry (DSC) was conducted using DSC Q20 V24.9 Build 121 (TA instruments). The DSC experiment was performed for the temperature range of −90 to 200 °C with a heating rate of 10 °C min^−1^. The FT‐IR spectra were recorded using an INVENIO‐R spectrometer (Bruker Optics GmbH) equipped with a diamond attenuated total reflection accessory (Golden Gate, Specac). Each spectrum, recorded as the average of 256 scans with a resolution of 2 cm^−1^, was collected from 4000 to 500 cm^−1^. Deconvolution of the FT‐IR peaks was performed by considering Gaussian peaks with several iterations to obtain the best‐fit Gaussian peak. Raman spectroscopic analyses were conducted using a DXR2xi Raman microscope (Thermo Fisher Scientific) with a 532 nm laser source.

### Electrochemical Characterization

The dielectric relaxation spectroscopy (DRS) measurements were conducted by Novocontrol GmbH Concept 40 broadband dielectric spectrometer on a series of i‐SPIDER films with a thickness of 200 µm. The i‐SPIDER films were prepared by sandwiching between freshly polished brass electrodes with two different diameters (top electrode: 15 mm, bottom electrode: 30 mm) to make a parallel plate capacitor cell. The sandwiched films were positioned in a Novocontrol GmbH Concept 40 broadband dielectric spectrometer having a Quatro Cryosystem sample chamber with a vacuum‐isolated cryostat and nitrogen line. These measurements were conducted using an AC voltage with an amplitude of 0.1 V over a frequency range of 10^−1^–10^−7^ Hz in all experiments. DRS curves were collected in isothermal frequency sweeps over a temperature range of 120 to −70 °C every 10 K.

### Actuation Characterization of i‐SPIDER Actuator

The measurement system used to analyze the actuation performance of a series of i‐SPIDER actuators was operated at various voltage with a sinusoidal and square waveform generated by a function generator (33500B, Agilent Technologies, Inc.). The actuators were fixed to a measuring holder with two separate copper electrodes. The custom‐built actuator measurement system consisting of a CCD camera, laser sensor (optoNCDT 1700–10, Micro‐Epsilon), force sensor (programmable x, y, and z‐axis stage at 0.1 µm resolution, FS1M‐0.1N, NanoControl), DC power supply, function generator, oscilloscope (DPO3052, Tektronix), and PC was analyzed for actual bending motion, displacement, and blocking force. The bending strain (*S*) and strain rate (*S_r_*) were calculated from the measured displacement according to the following equation:

where *δ* is the actuation displacement*, h and L_f_* are the thickness and free length of the actuator, respectively, and *f* is the frequency of applied AC voltage. In this study, a force sensor is used as the capacitance‐type sensor (FS1M‐0.1N, NanoControl). The force sensor is calibrated using a “weight”, and the output of the sensor amplifier and the voltage of the capacitance sensor are measured using a three‐point reference “weight”. The sensitivity is determined by linear approximation. As the force sensor used with a series of i‐SPIDER actuators is an important thing where 0 V is important at no load, the positions and conditions of the mechanical instrument reset the 0 V offset to an estimate before the measurement. The results of blocking force in this study are the offset measured with a minimum of three‐actuator devices and are the mean value measured within a 1% error range.

### Design and Fabrication of Arachnid‐Inspired i‐SPIDER Robot

The arachnid‐inspired soft robotic application experiments using the i‐SPIDER robot were conducted by fabricating bending actuators with i‐SPIDER (60/58). To mimic insect behavior, an artificial spider web was vibrated using the i‐SPIDER robot, which was designed to resemble a real spider. The robot consisted of eight i‐SPIDER actuators formed via spray coating, patterned as spider legs, with each leg connected to the central body through silver wires (0.05 mm). The vibration of the artificial spider web was controlled by applying an operating voltage through a function generator (33500B, Agilent Technologies, Inc.), with the applied voltage strength affecting the web vibration, as verified through experimentation (Figure 5a). To manufacture the i‐SPIDER robot with different leg angles, six i‐SPIDER legs were attached using polyethylene terephthalate (PET) and silver paste, with the silver wire serving as a common electrode for each leg. Objects weighing from 26.5 to 188 mg were prepared for lifting performance experiments by connecting up to seven styrofoam balls with an average weight of 26.5 mg and a diameter of 1 cm. Crawling locomotion experiments were conducted by assembling crawling robots and a locomotive i‐SPIDER by attaching PET legs to the actuator body using silver paste, with ionic PEDOT:PSS electrodes coated on both sides of the i‐SPIDER body connected to a function generator using silver wires.

## Conflict of Interest

The authors declare no conflict of interest.

## Supporting information



Supporting Information

Supplemental Movie 1

Supplemental Movie 2

Supplemental Movie 3

Supplemental Movie 4

Supplemental Movie 5

## Data Availability

The data that support the findings of this study are available from the corresponding author upon reasonable request.
