# Supplementary material for: Ions‐Silica Percolated Ionic Dielectric Elastomer Actuator for Soft Robots
Source: Adv Sci (Weinh). 2023 Oct 4;10(32):2303838. doi: 10.1002/advs.202303838 (PMC10646257; doi:10.1002/advs.202303838)
Supplement: Supplementary file 1 — Supporting Information [file ADVS-10-2303838-s006.pdf]

ADVANCED SCIENCE
Open Access

Supporting Information

for *Adv. Sci.*, DOI 10.1002/advs.202303838

Ions-Silica Percolated Ionic Dielectric Elastomer Actuator for Soft Robots

Hanbin Choi, Yongchan Kim, Seonho Kim, So Young Kim, Joo Sung Kim, Eseudeo Yun, Hyukmin Kweon, Vipin Amoli, U. Hyeok Choi*, Hojin Lee* and Do Hwan Kim**

Copyright WILEY-VCH Verlag GmbH & Co. KGaA, 69469 Weinheim, Germany, 2018.

Supporting Information

Ions-Silica Percolated Ionic Dielectric Elastomer Actuator for Soft Robots

Hanbin Choi,[#] Yongchan Kim,[#] Seonho Kim,[#] So Young Kim,[#] Joo Sung Kim, Eseudeo Yun, Hyukmin Kweon, Vipin Amoli,[*] U Hyeok Choi,[*] Hojin Lee,[*] Do Hwan Kim[*]

H. Choi, S. Y. Kim, J. S. Kim, H. Kweon
Department of Chemical Engineering, Hanyang University, Seoul 04763, Republic of Korea

Y. Kim, E. Yun
School of Electronic Engineering, Soongsil University, Seoul 06978, Republic of Korea

S. Kim
Department of Polymer Science and Engineering and Program in Environmental and Polymer Engineering, Inha University, Incheon 22212, Republic of Korea

Prof. V. Amoli
Department of Sciences and Humanities, Rajiv Gandhi Institute of Petroleum Technology, Amethi 229304, India
E-mail: vamoli@rgipt.ac.in

Prof. U. H. Choi
Department of Polymer Science and Engineering and Program in Environmental and Polymer Engineering, Inha University, Incheon 22212, Republic of Korea
E-mail: uhyeok@inha.ac.kr

Prof. H. Lee
School of Electronic Engineering, Soongsil University, Seoul 06978, Republic of Korea
School of Information Communication Convergence Technology, Soongsil University, Seoul 06978, Republic of Korea
E-mail: hojinl@ssu.ac.kr

Prof. D. H. Kim
Department of Chemical Engineering, Hanyang University, Seoul 04763, Republic of Korea
Institute of Nano Science and Technology, Hanyang University, Seoul 04763, Republic of Korea
Clean-Energy Research Institute, Hanyang University, Seoul 04763, Republic of Korea.
E-mail: dhkim76@hanyang.ac.kr

([#]These authors contributed equally to this work)

Keywords: soft robot, ionic electroactive polymer actuator, ions-silica percolated ionic dielectric elastomer, electrode polarization, electromechanical conversion

Supplementary Note S1: The correlation between the material characteristics and actuator performance in electrically driven soft actuators.

From the viewpoint of application to low-power miniaturized soft robotics, the improvement in the performances of the soft actuator increases the possibility of introduction. That is, next-generation soft actuators are being researched in the direction of improving both strain and force, which are the main performance indicators of actuators.[1, 2] The performances of electrically driven soft actuators, such as EAP and i-EAP, to convert the electrical source into mechanical work are closely related to the characteristics of their active materials. The relationship between these performances, such as strain (*S*), force (*F*), and electromechanical energy density (E_m), and material characteristics are given by the Maxwell stress tensor:[3, 4]

$$\mathrm{S} = \frac{\varepsilon_0 \varepsilon E^2}{Y},\ \mathrm{F} = \mathrm{Y}\varepsilon_0 \varepsilon E^2,\ E_m = \frac{(\varepsilon_0 \varepsilon)^2 E^4}{2Y} = \frac{YS^2}{2} \qquad (1)$$

where ε_0 is the vacuum permittivity, ε is the dielectric constant of the active material, *Y* is Young's modulus of the active material, and *E* is the applied electric field. First, actuator strain is inversely proportional to Young's modulus but has a proportional relation to the applied electric field and dielectric property. Conversely, for actuator force, all factors in the electric field, dielectric property, and Young's modulus show a proportional relation. In this regard, the mechanical property shows an inescapable conflicting effect on the strain and force in the soft actuators. That is, material approaches to simultaneously improve the strain and force of soft actuators is to apply a high electric field or use a material with a higher dielectric constant. However, the approach of increasing the electric field has limitations due to the need for an additional external power source to implement miniaturized soft robotic applications for the human-machine interface. Therefore, the strategy to improve the dielectric constant of the active layer is suitable as an approach that can improve all main actuator performances while compensating for the negative effect of the mechanical property on the actuator strain.

Supplementary Note S2: Microstructural characterization of a series of i-SPIDER films.

An investigation of the microstructural characteristics in the hybrid composite materials can serve to eliminate unwanted structural effects that hinder clarifying the interpretation of the electrode polarization effect for the enhanced main performances of i-SPIDER actuators. The field-emission scanning electron microscope (FE-SEM) image of the

i-SPIDER films (60 wt% Φ_{IL}) represents the well-dispersed silica microstructures in the i-SPIDER films. Also, energy-dispersive X-ray (EDX) spectroscopy elemental maps for C (mainly coming from TPU matrix), Si (coming from silica microstructure), and F (coming from IL) support IL-confined morphology of the silica microstructures in i-SPIDER films.[5]

The microstructures of these i-SPIDERs were analyzed using the X-ray diffraction (XRD), differential scanning calorimeter (DSC), and hydrogen bonding index (silica microparticle-free i-EAPs were used as reference) as shown in Figures 2, 3 and 4, respectively. In TPU, hard segments form well-organized domains that can serve as a physically crosslinked gel network, whereas the soft segments comprise the nanoscale free volume of the gel network. Such a two-segment conformation can be an ideal structure to simultaneously enhance mechanical and electrical properties. The broad crystalline XRD reflection at $2\theta \approx 19.79°$ (*d*-spacing: 4.48 Å) in pristine TPU is resulted from the well-organized domains of hard segments.[6, 7] After incorporating the ILs (20, 40, and 60 wt% Φ_{IL}) into the TPU matrix, hereafter referred to as i-EAP (20, 40, and 60), the relative intensity of crystalline XRD peak at 19.79° decreases. And a new peak appears at 12.1° (*d*-spacing: 7.2 Å) with increasing ionic concentration, which clearly indicates the intercalation of ions in TPU hard segments (Figure S2a). On the other hand, for the i-SPIDERs (60/19, 37, and 58), the absence of new peaks in the XRD patterns of the i-SPIDERs suggests the amorphous nature of the silica microstructure (Figure S2b). In this respect, DSC curves show the inner microstructure change of TPU matrix upon the IL and silica microparticles incorporation (Figure S3). With increasing the IL concentration in TPU, the glass transition temperature (T_g) of i-EAPs exhibits a dramatic shift toward lower temperature (Figure S3a). This indicates that there is a plasticizing effect due to the formation of ion pairs, which can interrupt the interaction between the TPU chains. On the other hand, the silica microstructures present in the soft segment in the TPU matrix have no effect on the T_g of the i-SPIDERs (60 wt% Φ_{IL}) (Figure S3b). This shows that the similar plasticizing effect by ionic liquids in i-EAP is also exhibited in i-SPIDERs, supporting that the microstructure of the TPU matrix is preserved in i-SPIDERs. In addition, as the hydrogen bond index (ratio of the peak area of hydrogen-bonded C=O groups/free C=O groups in the TPU chain) decreased according to the ion contents of i-EAP, the plasticizing effect was additionally confirmed by the reduction of hydrogen bonding between the C=O groups of TPU soft segments by the ILs (Figure S4a). In the case of i-SPIDERs introduced with silica microstructures, the significant increase in the hydrogen bond index of i-SPIDERs reflects the existence of new hydrogen bonding between the silica-surface silanol groups and the C=O groups of TPU chain (Figure S4b). Notably, as the silica contents increase, the hydrogen bond

index of the TPU chain decreases due to additional hydrogen bonding between the surface silanol groups and ILs, which means that more IL-confined regions are formed in i-SPIDER films. Consequently, without any changes in the TPU-characteristics XRD patterns and thermal properties in i-SPIDERs, the increased hydrogen bond index for the TPU chain suggests that the silica microstructures exist in the soft segments of the TPU matrix.

Supplementary Note S3: Understanding dielectric relaxation characteristics for interpretation of electrode polarization of the i-SPIDER films.

Measurements of the ion conductivity and dielectric constant are performed as a function of frequency and temperature for interpreting electrode polarization effect. Figure S6 displays the frequency dependence of dielectric permittivity (ε'), dielectric derivative spectra (ε_{der}) calculated via Supplementary Equation 2, and in-phase conductivity normalized by DC conductivity values (σ'/σ_{DC}), superimposed using time-temperature superposition (TTS) principle and frequency shift factor a_T.

$$\varepsilon_{\mathrm{der}} = -\frac{\pi}{2} \cdot \frac{\partial \varepsilon'(\omega)}{\partial \ln\omega} \tag{2}$$

This allows for the construction of master curves for the i-SPIDER (60/58), as a representative example, at a reference temperature (T_{ref} = 303 K), as shown in Figure S6. TTS was found to work well over the frequency range, and the temperature dependence of a_T follows the Williams-Landel-Ferry (WLF) equation (Figure S7 and Equation S4).[8, 9] The dielectric loss derivative spectra (ε_{der}) show two relaxation processes: segmental relaxation and ion rearrangement. The in-phase part of conductivity (σ') approaches to DC conductivity (σ_{DC}) after ion motion becomes diffusive at the time scale for conduction (See the yellow horizontal dashed line in Figure S6). The dielectric permittivity function (ε') significantly increases to the dielectric constant of electrode polarization (ε_{EP}) (See the dark green horizontal dashed line in Figure S6) at low frequencies, where the transporting ions have enough time to polarize at the blocking electrodes during cycle resulting in complete electrode polarization at frequency of electrode polarization (f_{EP}) (See the light green vertical dashed line in Figure S6). This finding is consistent with a decrease in σ'/σ_{DC} at low frequencies as the polarizing ions reduce the field experienced by the transporting ions. Typical i-EAP actuators have been operated in the bandwidth range blow 1 kHz. Thus, the fact that transporting ions fully polarize at frequencies below 10^4 Hz suggests that our i-SPIDER actuators likely depend on the polarization of ions occurring at lower frequencies.

Polarization time scale (τ_{EP}), which is the reciprocal of f_{EP}, increases with increasing silica content (Φ_{SiO_2}), which in turn indicates that incorporation of IL-confined silica microstructure leads to the delay of electrode polarization in i-SPIDERs (60 wt% Φ_{IL}), as shown in Figure S9. The similar trend (increasing τ_{EP} or slowing down electrode polarization) is also observed in other i-SPIDERs using 20 and 40 wt% Φ_{IL}, depending on the presence of silica microstructure (Φ_{SiO_2} = 0, 19 wt%) (Figure S10a). Additionally, the time scale of polarization (or charging) can be analytically considered for a simple equivalent circuit model of resistor and capacitor, which can be described as:

$$\tau_{\mathrm{EP}} = RC \tag{3}$$

where C is the capacitance and R is the resistance of the equivalent circuit. The charge (Q) built up within the Stern layer is the product of C and voltage (V); i.e., $Q = CV$. Resistance depends on the conductivity and geometrics ($R = L/\sigma_{DC} A$), where L and A are the thickness of the active layer and surface area of the electrode, respectively. Therefore, the charge density (Q/A) has a close relationship with the time scale (τ_{EP}) for complete polarization at the electrode. That is, an increase in the charge density due to ion polarization at the interface between the i-SPIDER and electrode enhances the electro-mechanical conversion in the actuators, which is induced by the volumetric change in the i-SPIDER films as the result of ion accumulation at the interface.[10, 11]

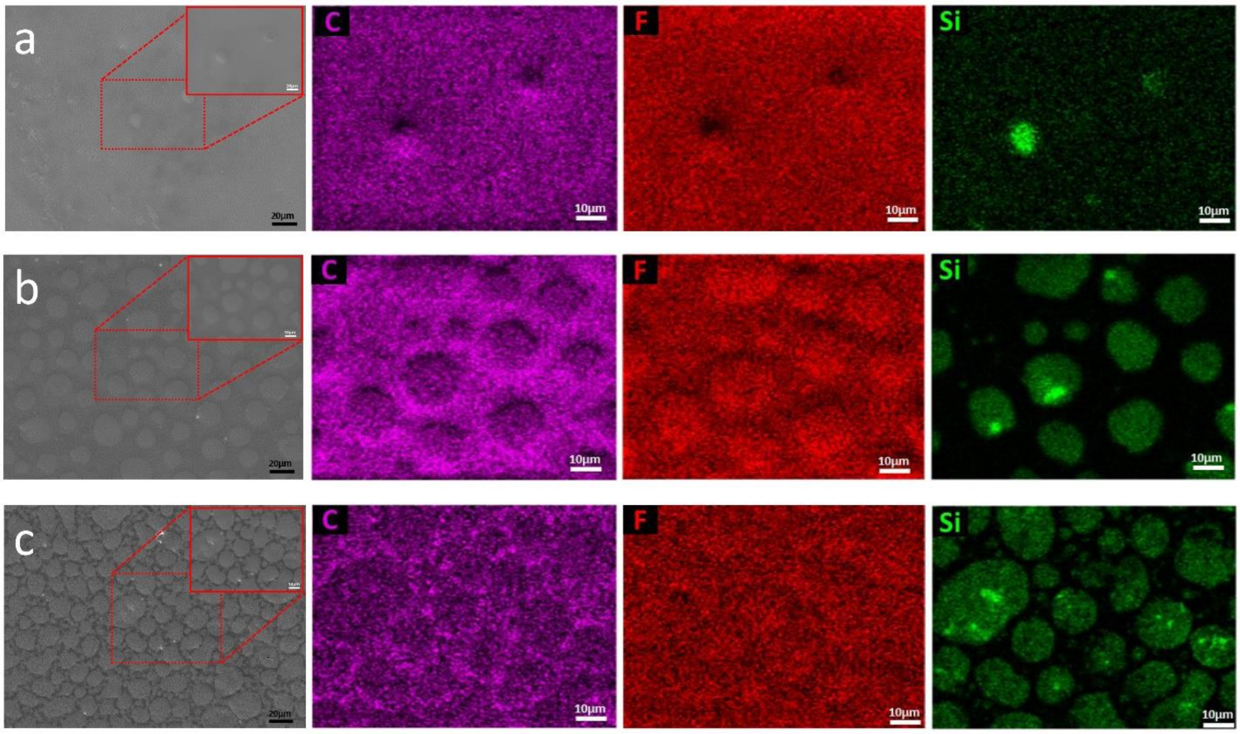

Figure S1. FE-SEM image and EDX elemental maps (corresponding to FE-SEM image shown in inset, red box) for C, F, and Si. (a) i-SPIDER (60/19). (b) i-SPIDER (60/37). (c) i-SPIDER (60/58). Scale bars, 20 µm (black); 10 µm (white).

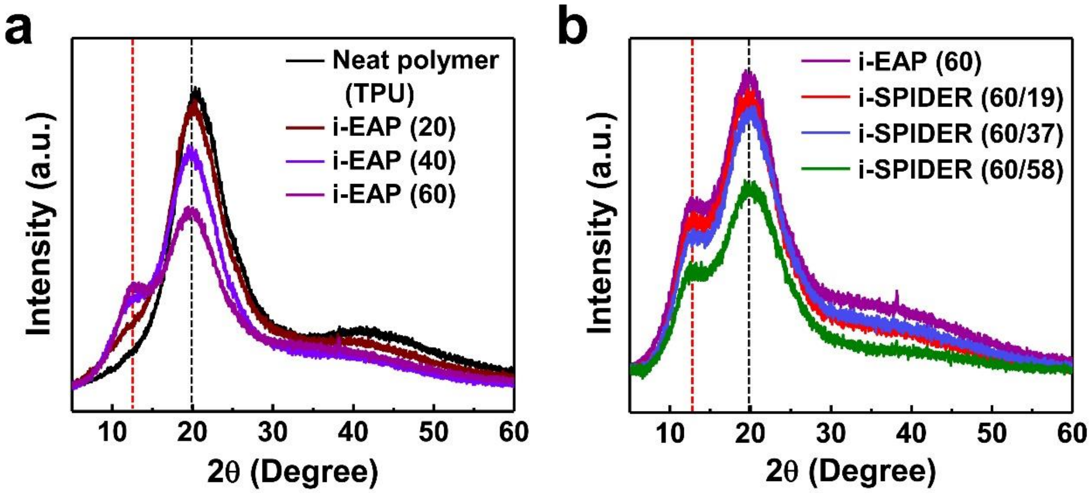

Figure S2. XRD patterns of (a) neat polymer (TPU) film and i-EAP films with different contents of IL and (b) i-SPIDER films (60 wt% Φ_{IL}) with different silica contents.

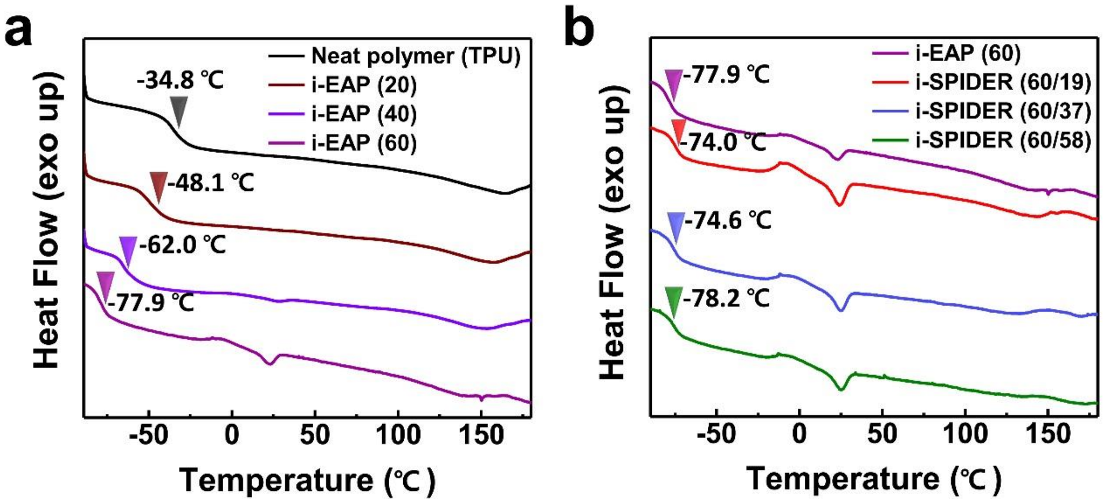

Figure S3. DSC thermogram of (a) neat polymer (TPU) film and i-EAP films with different contents of IL and (b) i-SPIDER films (60 wt% Φ_{IL}) with different silica contents.

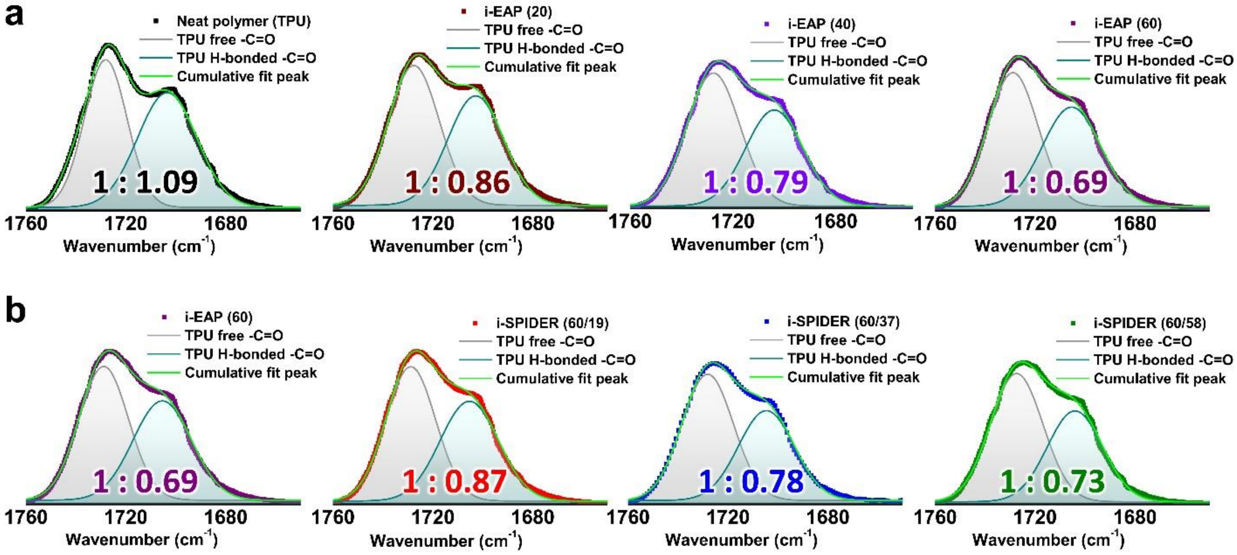

Figure S4. The hydrogen bond index in (a) neat polymer (TPU) film and i-EAP films with different contents of IL and (b) i-SPIDER films (60 wt% Φ_{IL}) with different silica contents.

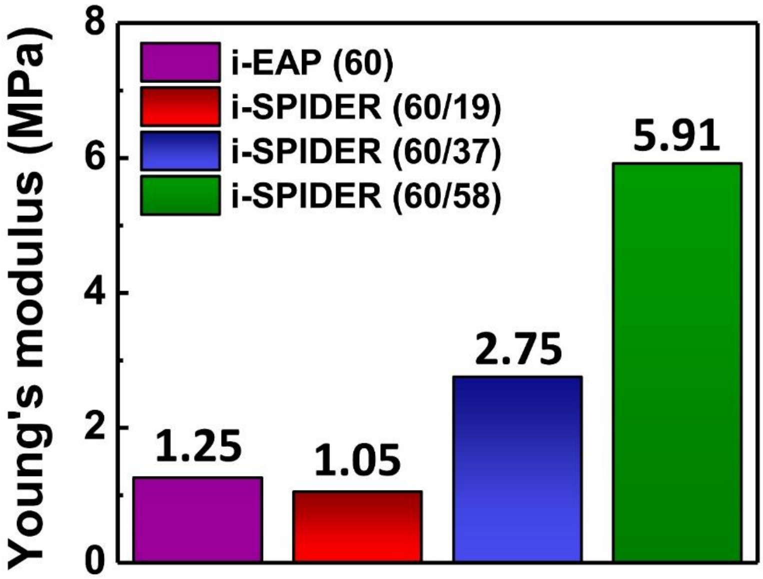

Figure S5. Young's modulus of a series of i-SPIDER films (60 wt% Φ_{IL}) with different silica contents. These mechanical properties are derived from stress-strain curve, as shown in Figure 1e, Main manuscript.

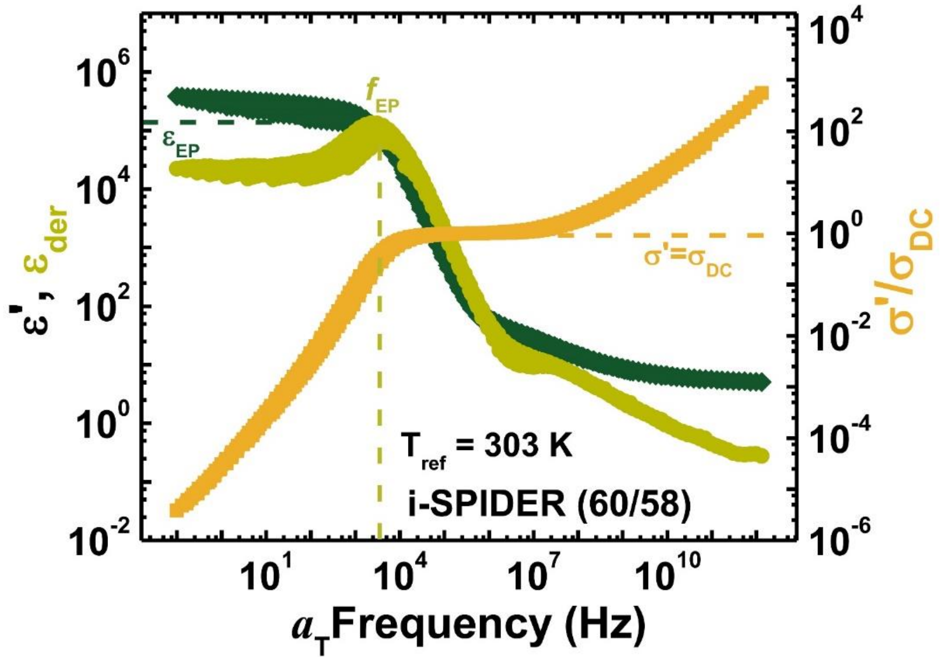

Figure S6. Master curves of dielectric permittivity (ε' (a_T), left axis), dielectric derivative spectra (ε_{der} ($a_T f$), left axis), and in-phase conductivity normalized by DC conductivity (σ'/σ_{DC} ($a_T f$), right axis) for i-SPIDER (60/58) at reference temperature (T_{ref} = 303 K).

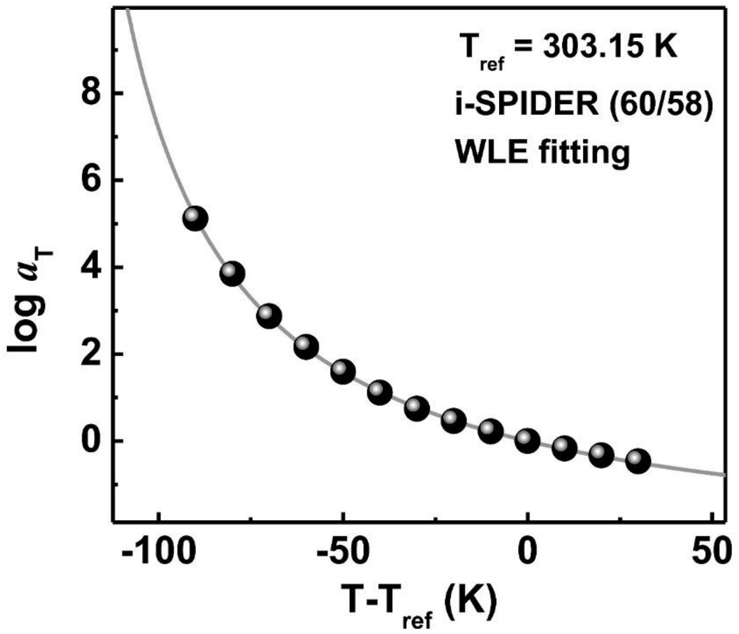

Figure S7. Dielectric shift factor (a_T) for master curves of $\varepsilon'(a_T f)$, $\varepsilon_{der}(a_T f)$, and $\sigma'/\sigma_{DC}(a_T f)$ data of i-SPIDER (60/58). The solid line is a fit of the WLF equation (Equation S4) at reference temperature (T_{ref} = 303 K). Time-temperature superposition (TTS) and frequency shift factors (a_T) allow to construct master curves. The temperature dependence of a_T follows the Williams-Landel-Ferry (WLF) equation:[8, 9]

$$\log(a_T) = -\frac{C_1(T-T_{\mathrm{ref}})}{C_2+T-T_{\mathrm{ref}}} = -\frac{C_1(T-T_{\mathrm{ref}})}{T-T_0} \quad (4)$$

wherein C_1 and C_2 ($T_{ref} - T_0$) are constants, and T_0 is the Vogel temperature.

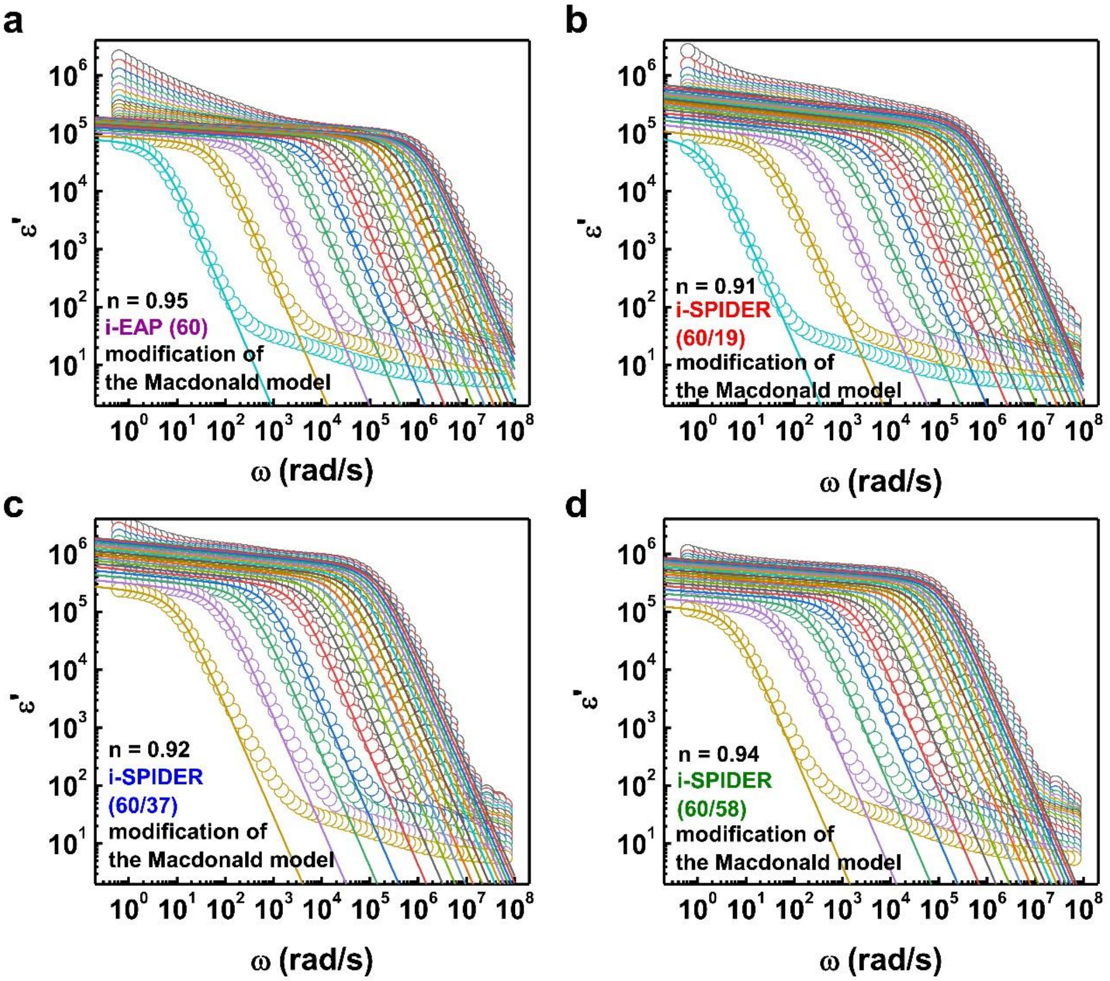

Figure S8. Dielectric permittivity spectra (*ε'(ω)*, open symbol) of (a) i-EAP (60), (b) i-SPIDER (60/19), (c) i-SPIDER (60/37), and (d) i-SPIDER (60/58) at various temperature (a and b: -60 ℃ ~ 120 ℃, c and d: -50 ℃ ~ 120 ℃). Solid lines are fits to empirical modification of the Macdonald model (Supplementary Equation 5) with values of n = 0.95, 0.91, 0.92, and 0.94, respectively. The electrode polarization frequency, ω_{EP} (= $1/\tau_{EP}$) is calculated using the empirical modification of the Macdonald model,[12] which is given by

$$\varepsilon_{EP}^{*}(\omega) = \frac{\Delta\varepsilon_{EP}}{(i\omega/\omega_{EP})^{1-n} + i\omega/\omega_{EP}} \qquad (5)$$

wherein $\Delta\varepsilon_{EP}$ is the dielectric increment of electrode polarization, ω_{EP} ($2\pi f_{EP}$) is the characteristic frequency of electrode polarization, and the exponent n is connected with electrode roughness.

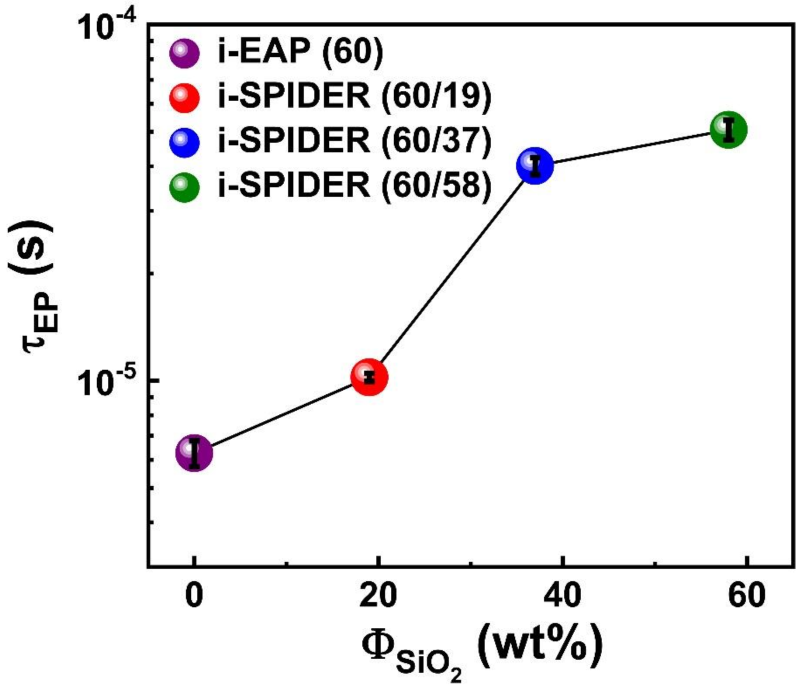

Figure S9. Compositional variation of Φ_{SiO_2} in the time scale of electrode polarization (τ_{EP}) of a series of i-SPIDERs (60 wt% Φ_{IL}). Error bars indicate standard deviation.

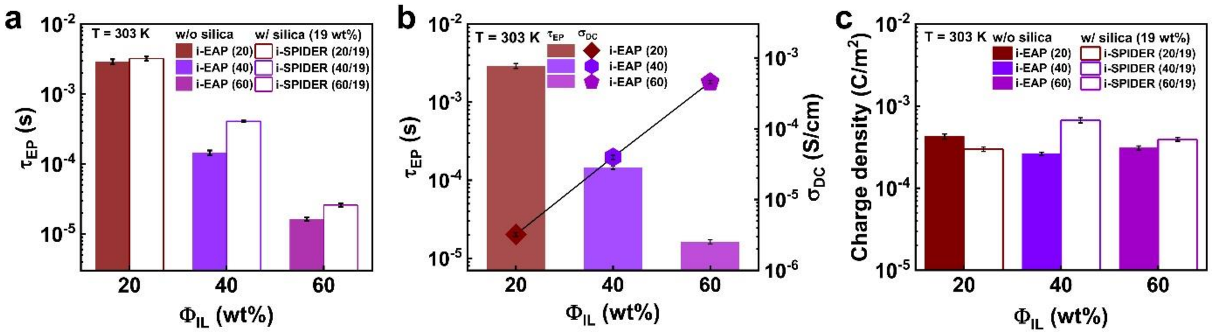

Figure S10. (a) Compositional variation of Φ_{IL} in τ_{EP} for i-EAPs (filled bar symbols) and i-SPIDERs with fixed silica content (19 wt% Φ_{SiO_2}) (open bar symbols). (b) Compositional variation of Φ_{IL} in τ_{EP} (filled bar symbols, left axis) and DC conductivity (σ_{DC}) (scattering symbols, right axis). (c) Compositional variation of Φ_{IL} in the charge density at the electrode for i-EAPs (filled bar symbols) and i-SPIDERs with fixed silica content (19 wt% Φ_{SiO_2}) (open bar symbols). In the i-SPIDER with 40 wt% Φ_{IL}, the charge density increased by a factor of 2, compared to that in the i-EAP with the same IL content. Error bars indicate standard deviation.

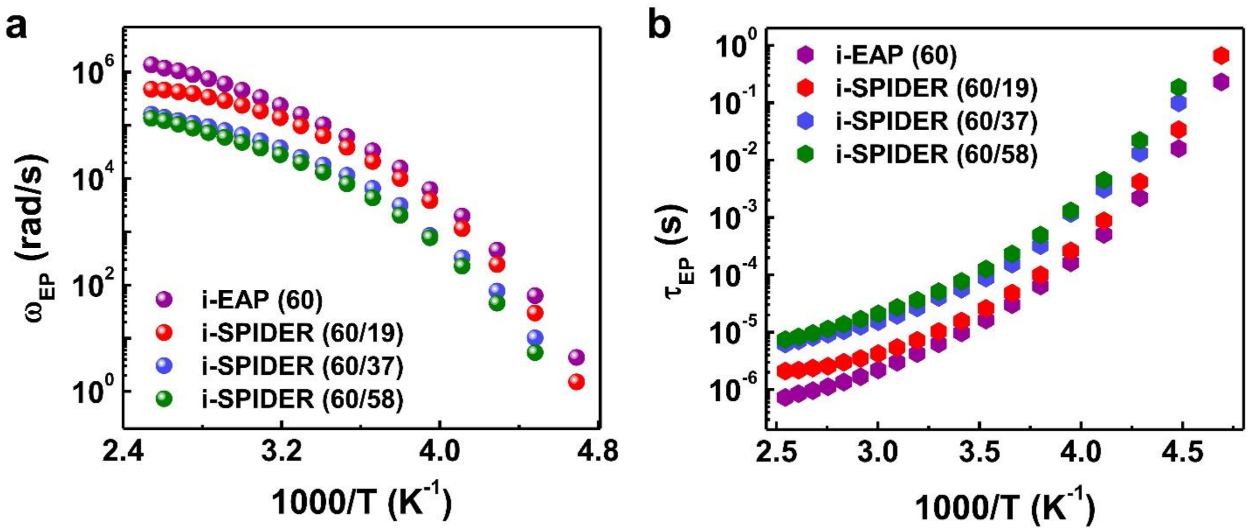

Figure S11. Temperature dependence of (a) angular frequency (ω_{EP}) and (b) time scale of electrode polarization (τ_{EP}) for a series of i-SPIDERs (60 wt% Φ_{IL}) with various silica contents.

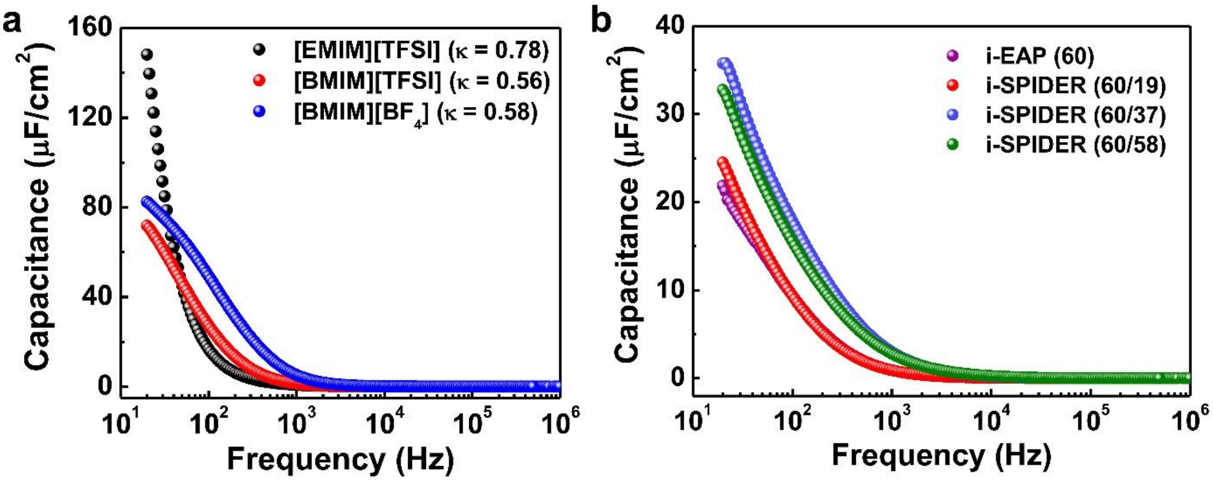

Figure S12. Capacitance-frequency characteristics of (a) the pure ionic liquids with different ionicity (κ) and (b) a series of i-SPIDER films (60 wt% Φ_{IL}) under 1V AC bias.

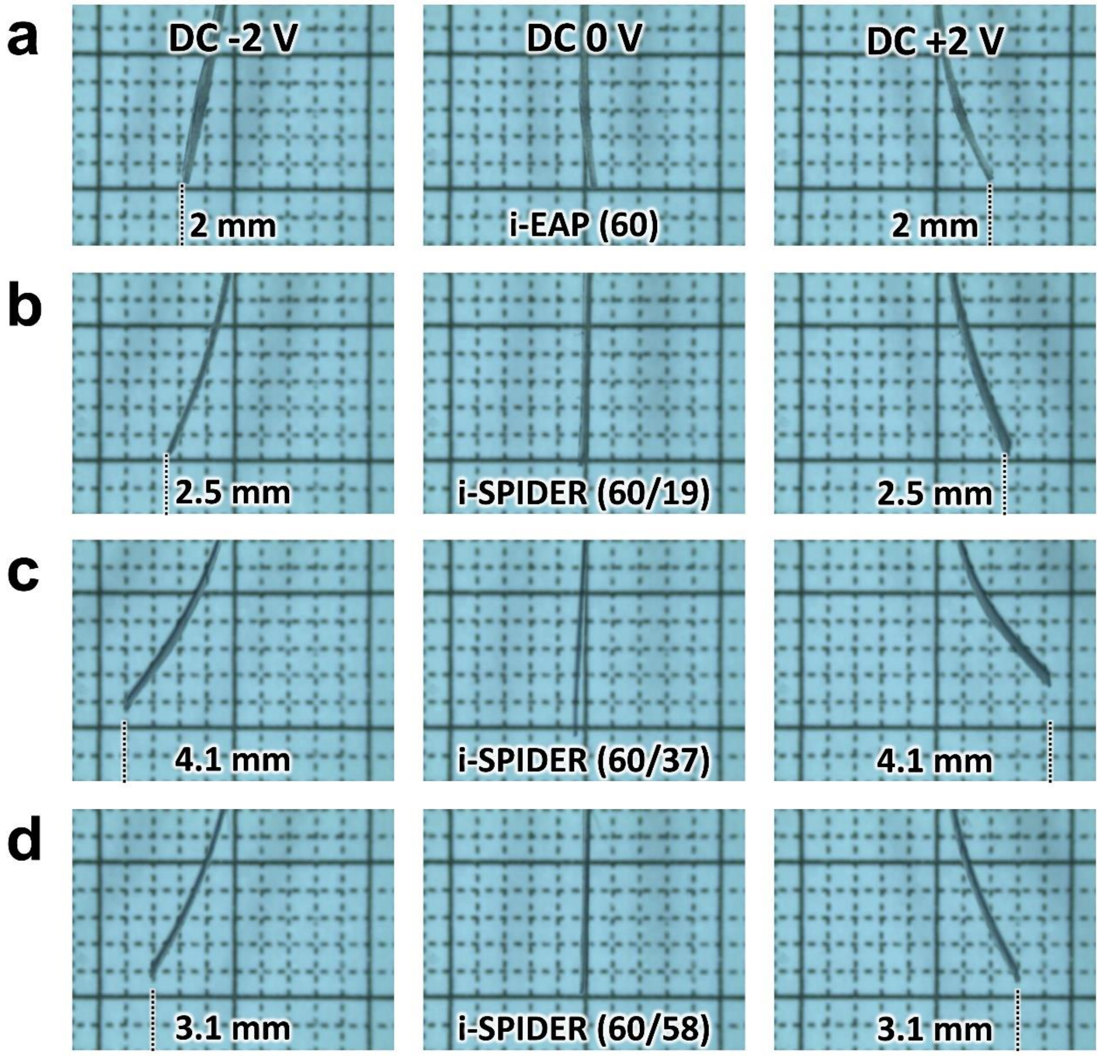

Figure S13. Photograph images of the i-SPIDER actuators with their corresponding degree of bending motion depending on (a) i-EAP (60), (b) i-SPIDER (60/19), (c) i-SPIDER (60/37), and (d) i-SPIDER (60/58). These actuators contained ionic PEDOT:PSS electrode layers and were operated at a dc voltage of ±2 V. The degree of bending motion of i-EAP (60)-based actuator is 2.0 mm. The degree of bending motion of i-SPIDER actuators is 2.5, 4.1, and 3.1 mm with different silica contents (19, 37, and 58 wt% Φ_{SiO_2}), respectively.

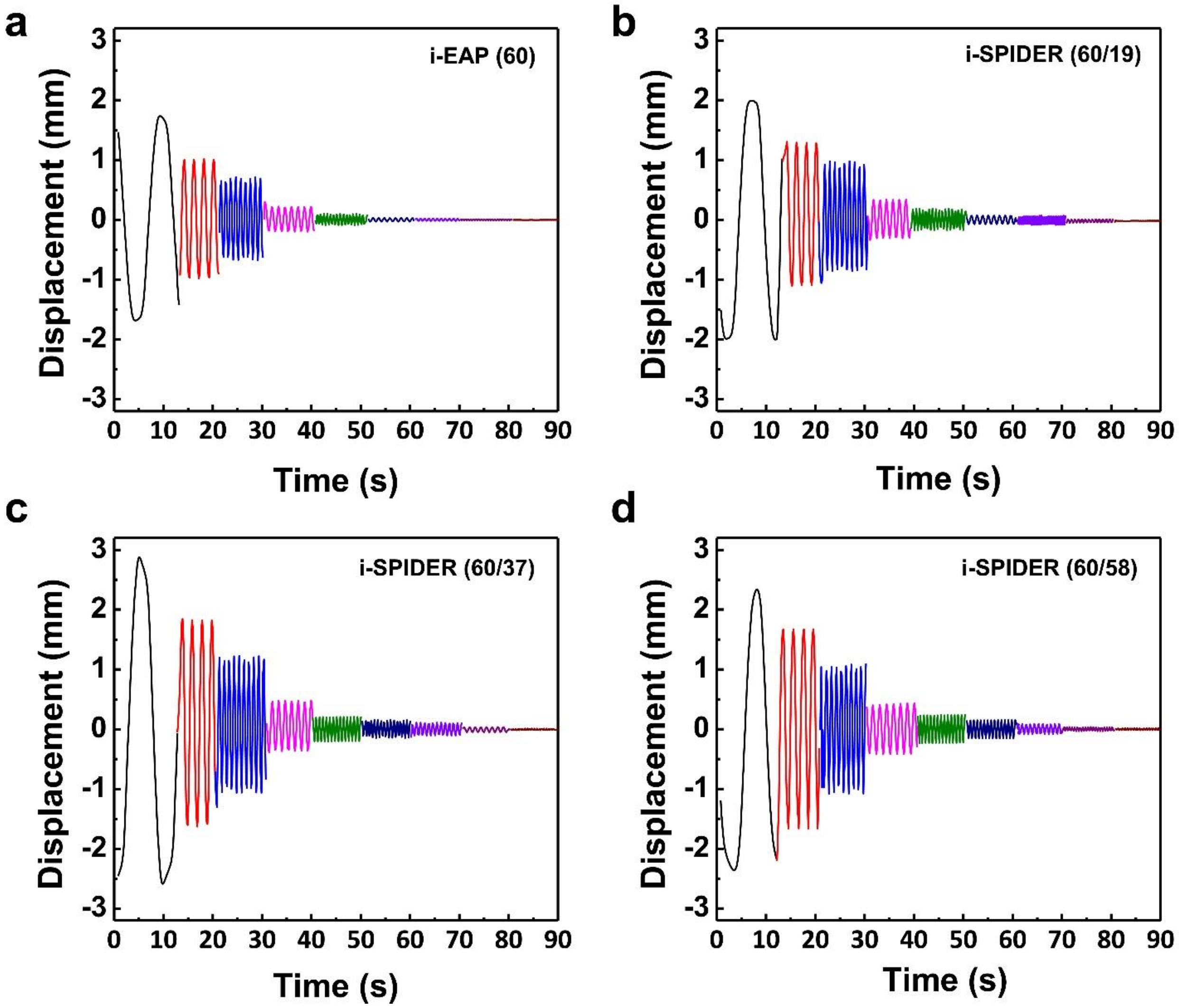

Figure S14. Displacement characteristics of the i-SPIDER actuators depending on types of active layer in terms of frequencies; (a) i-EAP (60), (b) i-SPIDER (60/19), (c) i-SPIDER (60/37), and (d) i-SPIDER (60/58). For measuring displacement, a light output source of laser sensor with wavelength of 670 nm, power of 1mW was utilized, and the distance between actuator and laser sensor was 30 mm.

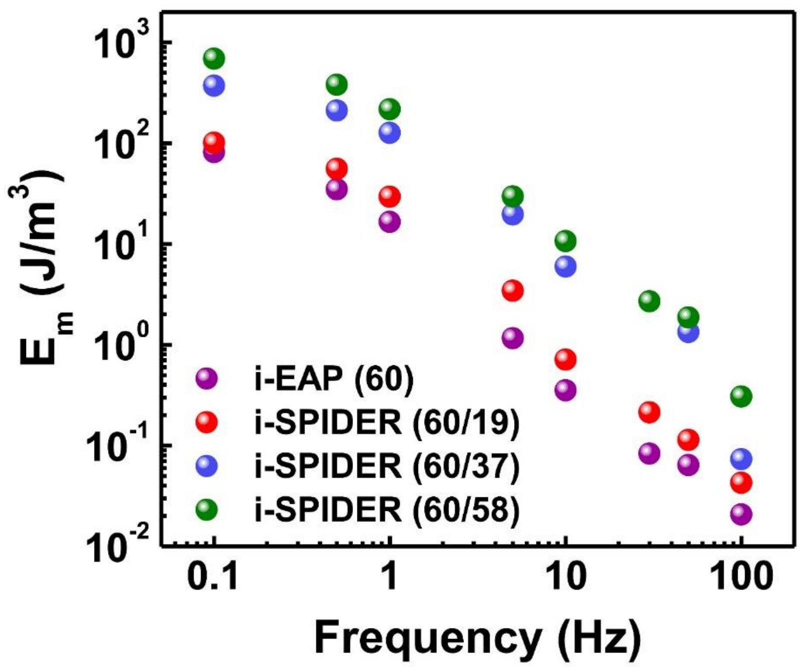

Figure S15. Electromechanical energy density (E_m) derived from strain performance (Fig. 4b, Main manuscript and Equation S1) for a series of i-SPIDER (60 wt% Φ_{IL}) actuators with different silica contents.

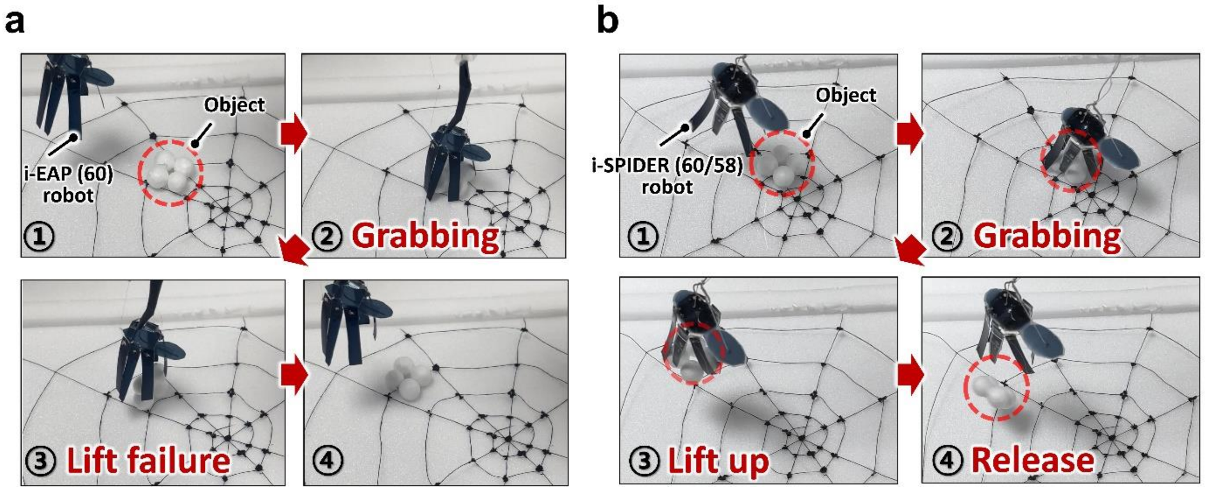

Figure S16. Photographs of the comparison between (a) the i-EAP (60) robot and (b) the proposed i-SPIDER (60/58) robot for a series of object transfers, including grabbing, lifting up, and releasing the object.

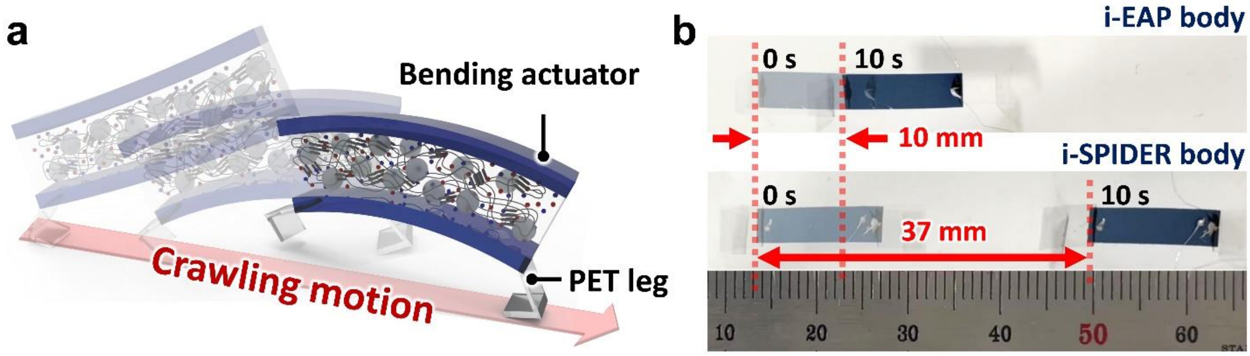

Figure S17. Comparison of locomotion characteristics of the crawling robots. (a) Schematic picture of a crawling robot composed of a bending actuator and PET legs. (b) Photographs comparing the crawling robots using i-EAP body and i-SPIDER body as the bending actuator.

Table S1. List of pros and cons of properties of main materials (ionic EAP and electrical EAP) for the electrically driven actuator.

Type	Pros and cons	
	Advantages	Disadvantages
Ionic EAP	- Low operating power<br>(≤ a few V)<br>- Relative high response<br>- Large strain<br>- High softness	- Low electromechanical energy density<br>- low actuation force
Electrical EAP	- High electromechanical<br>energy density<br>- High actuation speed<br>- Large actuation force	- High operating power (≥ a few kV)<br>- Relatively low softness

Table S2. Presentation of mass ratios of crucial tri-component which constitute the ions-silica percolated ionic dielectric elastomers used in this study.

Sample	Mass ratio (TPU/IL/TEOS)	IL contents (wt%)	Silica contents (wt%)
i-EAP (20 wt%)	1.00/0.25/0.00	20	0
i-EAP (40 wt%)	1.00/0.67/0.00	40	0
i-EAP (60 wt%)	1.00/1.50/0.00	60	0
i-SPIDER (20/19 wt%)	1.00/0.25/0.24	20	19
i-SPIDER (40/19 wt%)	1.00/0.67/0.24	40	19
i-SPIDER (60/19 wt%)	1.00/1.50/0.24	60	19
i-SPIDER (60/38 wt%)	1.00/1.50/0.59	60	37
i-SPIDER (60/58 wt%)	1.00/1.50/1.40	60	58

Table S3. Comparison of bending strain and blocking force of the i-SPIDER actuator and most represent low-voltage driven i-EAP based actuators reported in the literature for the last 7 years.

Actuator type (active layer/electrode)	Young's modulus (MPa)	Blocking force (mN)	Bending strain[a] (%)	Input bias conditions	Ref.[b]
i-EAP actuator (i-SPIDER (60/58)/ PEDOT:PSS-DMSO-IL	5.9	1.06	1.52	±2 V and 0.1 Hz	This work
		0.77	0.86	±2 V and 1 Hz	
i-EAP actuator (IL-TPU/PEDOT:PSS-DMSO-IL)	1.2	0.46	0.64	±2 V and 0.1 Hz	15
i-EAP actuator (Zwitterion-IL-PVA/PEDOT:PSS)	6.1	0.1	0.35	±2 V and 0.1 Hz	21
i-EAP actuator (Zwitterion-Nafion/SWCNTs)	-	0.30	0.90	±2 V and 0.5 Hz	20
i-EAP actuator (IL-TPU/PEDOT:PSS-MWCNT)	82.18	0.5	0.65	±2.5 V and 0.1 Hz	22
i-EAP actuator (IL-PS/PEDOT:PSS-DMSO-IL)	100	1.5	0.42	±2 V and 1 Hz	14
i-EAP actuator (IL-CA-GN/PEDOT:PSS)	831	0.44	0.15	±2 V and 0.1 Hz	34
IPMC actuator (IL-Terpolymer/Pt nanoparitcle)	3.2	0.13	0.1	±1 V and 0.2 Hz	23
IPMC actuator (IL-Nafion/PEDOT:PSS-CTF)	48.56	2.05	0.38	±1 V and 0.1 Hz	25
IPMC actuator (IL-PVDF/Ni-MOF)	1,000	1.45	0.36	±3 V and 0.1 Hz	57
IPMC actuator (IL-Nafion/Pt)	-	0.5	0.59	±2 V and 0.1 Hz	18
IPMC actuator (IL-Nafion/Pt)	-	5	0.36	±2 V and 0.1 Hz	13

[a]The bending strains were calculated from the peak-to-peak displacement of each actuator.
[b]Reference number from manuscript.

Movie S1. Individual operating motion of each i-SPIDER leg of the arachnid-inspired soft robot.

Movie S2. Vibration motion of artificial spider web by leg movement of i-SPIDER robot.

Movie S3. Comparison of object manipulation of i-EAP (60) and i-SPIDER (60/58) robots.

Movie S4. Comparison of crawling distance between i-EAP and i-SPIDER body.

Movie S5. Crawling motion of locomotive i-SPIDER.

Supplementary References

1. R. Y. Wang, S. Jeong, H. Ham, J. Kim, H. Lee, C. Y. Son, M. J. Park, *Adv. Mater*. **2022**, *35*, 2203413.
2. M. Nan, F. Wang, S. Kim, H. Li, Z. Jin, D. Bang, C. S. Kim, J. O. Park, E. Choi, *Sens. Actuators B Chem*. **2019**, *301*, 127127.
3. N. Della Schiava, K. Thetpraphi, M. Q. Le, P. Lermusiaux, A. Millon, J.-F. Capsal, P.-J. Cottinet, *Polymers*. **2018**, *10*, 263.
4. T. Y. K. Ho, A. Nirmal, M. R. Kulkarni, D. Accoto, N. Mathews, *Adv. Intell. Syst.* **2022**, *4*, 2100061.
5. V. Amoli, J. S. Kim, E. Jee, Y. S. Chung, S. Y. Kim, J. Koo, H. Choi, Y. Kim, D. H. Kim, *Nat. Commun.* **2019**, *10*, 4019.
6. X. Chen, W. Wang, C. Jiao, *RSC advances*, **2016**, *6*, 92276.
7. J. I. Lee, H. Choi, S. H. Kong, S. Park, D. Park, J. S. Kim, S. H. Kwon, J. Kim, S. H. Choi, S. G. Lee, D. H. Kim, and M. S. Kang, *Adv. Mater.* **2021**, *33*, 2100321.
8. M. L. Williams, R. F. Landel, J. D. Ferry, *J. Am. Chem. Soc.* **1955**, *77*, 3701.
9. M. Lee, H. W. Gibson, T. Kim, R. H. Colby, U. H. Choi, *Macromolecules*, **2019**, *52*, 4240.
10. R. H. Colby, A. A. Kornyshev, *Soft Matter*, **2013**, *9*, 3767.
11. R. H. Colby, A. A. Kornyshev, *J. Phys. Condens. Matter*. **2013**, *25*, 082203.
12. D. Fragiadakis, S. Dou, R. H. Colby, J. Runt, *Macromolecules*, **2008**, *41*, 5723.
